# Ti_3_C_2_T_x_ Composite Aerogels Enable Pressure Sensors for Dialect Speech Recognition Assisted by Deep Learning

**DOI:** 10.1007/s40820-024-01605-z

**Published:** 2024-12-30

**Authors:** Yanan Xiao, He Li, Tianyi Gu, Xiaoteng Jia, Shixiang Sun, Yong Liu, Bin Wang, He Tian, Peng Sun, Fangmeng Liu, Geyu Lu

**Affiliations:** 1 State Key Laboratory of Integrated Optoelectronics, College of Electronic Science and Engineering, Jilin University, Changchun, 130012 People’s Republic of China; 2 School of Integrated Circuits, Tsinghua University, Beijing, 100084 People’s Republic of China; 3 International Center of Future Science, Jilin University, Changchun, 130012 People’s Republic of China

**Keywords:** Pressure sensor, Wearable sensor, Ti_3_C_2_T_x_ composite aerogel, Dialect speech recognition

## Abstract

Wearable pressure sensors capable of adhering comfortably to the skin hold great promise in sound detection. However, current intelligent speech assistants based on pressure sensors can only recognize standard languages, which hampers effective communication for non-standard language people. Here, we prepare an ultralight Ti_3_C_2_T_x_ MXene/chitosan/polyvinylidene difluoride composite aerogel with a detection range of 6.25 Pa-1200 kPa, rapid response/recovery time, and low hysteresis (13.69%). The wearable aerogel pressure sensor can detect speech information through the throat muscle vibrations without any interference, allowing for accurate recognition of six dialects (96.2% accuracy) and seven different words (96.6% accuracy) with the assistance of convolutional neural networks. This work represents a significant step forward in silent speech recognition for human–machine interaction and physiological signal monitoring.

**Supplementary Information:**

The online version contains supplementary material available at 10.1007/s40820-024-01605-z.

## Highlights

- Emphasized the innovation in both the material design and methodology between the sensing performance and mechanical properties.

- The composite aerogel pressure sensors exhibited low hysteresis (13.69%), wide detection range (6.25 Pa-1200 kPa), and cyclic stability to acquire stable and accurate pronunciation signals.

- Over 6888 and 4158 pronunciation signals were collected by the pressure sensor and utilized for training the convolutional neural network model, allowing for accurate recognition of six dialects (96.2% accuracy) and seven words (96.6% accuracy).

**Supplementary Information:**

The online version contains supplementary material available at 10.1007/s40820-024-01605-z.

## Graphical abstract

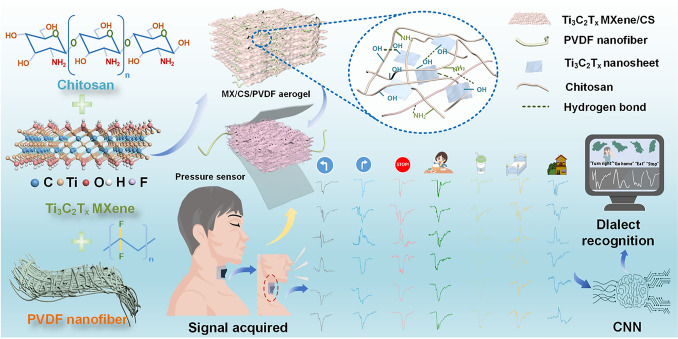

## Introduction

Spoken recognition as a branch of speech recognition can assist people with language barriers as well as human–computer interactions to express ideas and give instructions. The present spoken recognition involves the detection of sound waves directly, including spectral analysis, extraction and comparison of acoustic features, and acoustic texture analysis [1–3]. However, the direct detection approach is susceptible to interference by the transmission media, ambient noise, and the physiological state of the speakers. Speech recognition through mechanical sensors can avoid these defects by detecting the vibration of throat muscles based on the anatomical foundation of the throat during vocalization [4–7].

Wearable pressure sensors that can convert throat vibrations into visualized electrical signals have received widespread attention in detecting speech information [8–11]. Initially, speech recognition was mainly implemented by comparing the waveforms of electrical signals of throat vibrations captured using pressure sensors or tactile sensors [12, 13]. In addition, the pressure sensor can detect vibrations within the throat muscles and distinguish different pronunciations by simple signal processing, such as calculating the slope of signal peaks and comparing peak widths [14]. With the advancement of artificial intelligence (AI) technology, machine learning was introduced to build models for training and recognition of different pronunciations, particularly the combination of pressure sensors and machine learning [15–20]. Convolutional neural network (CNN) and support vector machine have frequently been introduced to identify the collected pronunciation signal for speech recognition [8, 21]. However, pressure sensors for speech recognition are currently restricted to identifying standard languages, which hampers effective communication for dialect speakers [5, 22]. For tone languages, the differences between dialect pronunciations are tone and pitch, which are generated by the throat muscles controlling the movement of the hyoid bone and cartilage. The elevation or depression of voice pitch is closely related to the contraction and relaxation of the throat muscles. The primary challenge in dialect recognition through pressure sensors with narrow-detection range and hysteresis lies in the difficulty in capturing the subtle and rapid vibrations of throat muscles during the vocalization process [23]. These factors place stringent demands on the pressure-sensing performance, such as low detection limit, high stability, and hysteresis characteristics.

To fulfill the requirements for speech recognition, Ti_3_C_2_T_x_ MXene has emerged as a promising candidate for wearable pressure sensors due to its adjustable layer spacing and superior conductivity [24–26]. However, pure Ti_3_C_2_T_x_ typically suffers from mechanical brittleness and oxidization, rendering it susceptible to collapse during repeated cycles [27]. To prevent sensitivity degradation under mechanical stimuli, compositing Ti_3_C_2_T_x_ layers with a nanostructured polymer matrix offers enhanced specific surface area and more contact points [28]. An aerogel structure with high porosity is essential for creating efficient electrical connections and increasing the compressibility of the sensor layer [29], resulting in changes in electrical conductivity when exposed to external pressure. Chitosan (CS), as a polysaccharide biopolymer, may substantially increase the degree of freedom in molecular movement, ultimately improving flexibility by forming robust hydrogen bonding between the biopolymer and Ti_3_C_2_T_x_ [30, 31]. Polyvinylidene difluoride (PVDF) short fibers, serving as a reinforcing phase, improve the durability of the aerogel by providing reversible deformation under high pressure as well [32–35]. Compared to the widely used conductive aerogel for pressure sensor, this polymer fibers reinforcement aerogel outperforms conductive aerogel by leveraging low density, excellent reversible deformation under high pressure for wide detection range, as well as low detection limit attributed to low compression modulus for the acquisition of small signals during speech recognition. Therefore, compositing polymers into Ti_3_C_2_T_x_-based aerogels is a feasible approach to achieving wearable pressure sensors with enhanced sensitivity and mechanical stability.

**Fig. 1:**
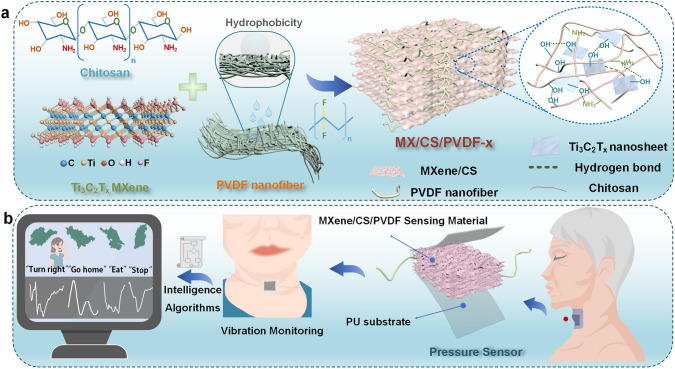
**a** Schematic preparation of MX/CS/PVDF aerogel. **b** Wearable Ti_3_C_2_T_x_-based aerogel pressure sensor for dialect speech recognition

Herein, we have fabricated wearable pressure sensors based on the laminar-like aerogel structure of Ti_3_C_2_T_x_ MXene/CS/PVDF composites (Fig. 1a). A CNN algorithm is adopted to manage the sensing information acquired by pressure sensors, leading to accurate dialect recognition (96.2% for six dialects and 96.6% for seven common vocabularies) that can satisfy the communication demand for dialect-speaking people (Fig. 1b). This work propels the development of pressure sensors for dialect recognition.

## Experimental

### Preparation of Ti_3_C_2_T_x_ MXene Nanosheet

Ti_3_C_2_T_x_ MXene nanosheets were synthesized by less violently etching the Ti_3_AlC_2_ (Jilin 11 Technology Co., Ltd., China) utilizing LiF/HCl mixed solution for removing the Al layer from the MAX phase. First, 2 g LiF (Aladdin, > 99.99%) was slowly dissolved in 80 mL 12 M HCl (Xilong Scientific Co., Ltd.) in a Teflon lining with vigorous stirring for 15 min at room temperature. 2 g Ti_3_AlC_2_ was then gradually added into the above mixed solution and reacted at 45 °C of water bath for 24 h. After etching, the resultant mixture was repeatedly centrifuged for 8 min at 8000 rpm until the pH of the supernatant reached 6 ~ 7. The clay mixture was ultrasonically dispersed in an ice bath at 350 W for 1 h. Finally, Ti_3_C_2_T_x_ MXene nanosheets were obtained by centrifugation at 10,000 rpm for 10 min.

### Preparation of PVDF Fibrous Membrane

PVDF fibrous membranes were prepared by solution electrospinning. The electrospinning precursor solution was 8 wt% PVDF (Solef 6010, Solvay S.A) in DMF and acetone mixture (7/3, w/w) and placed in 10 mL syringes with a 19 g blunt needle and controlled by programmed syringe pumps with 0.8 mL h^–1^ feed rate. 16 kV high voltage was provided by a high voltage power supply with a nozzle-to-collector distance of 18 cm. In the electrospinning process, the environment humidity and temperature were fixed at 58 ± 2%, 20 °C.

### Preparation of MX/CS/PVDF Aerogels

0.5 g above PVDF fibrous membrane was put into 500 mL deionized water and broken into suspension by a high-speed blender for 10 min. Then, 2.5 g CS (Aladdin, degree of deacetylation ≥ 75%) and 5 mL acetic acid (Xilong Scientific Co., Ltd., > 99.5%) were dissolved in the above mixture. After stirring for 20 min, the different weight of Ti_3_C_2_T_x_ MXene nanosheet was added into CS/PVDF suspension, and the weight ratios of Ti_3_C_2_T_x_ and CS were 0.5/1, 1/1, 1.5/1, 2/1, respectively.

Homogeneous aerogel precursor solutions were obtained by ultrasound treatment for 20 min. Then, 8 mL aerogel precursor was injected into 4 × 4 × 4 cm^3^ Teflon mold and was directly frozen from the bottom by liquid nitrogen. Subsequently, the mixture was freeze-dried in a vacuum lyophilizer (Beijing Biocool Co., Ltd., FD-1A-50) for 16 h. Herein, the sample was defined as MX/CS/PVDF-x (*x* = 0.5, 1, 1.5, 2), where x was represented as the ratio between Ti_3_C_2_T_x_ MXene and CS.

### Characterizations

The morphology of Ti_3_C_2_T_x_ MXene nanosheet, PVDF fibrous membrane, and MX/CS/PVDF-x (*x *= 0.5, 1, 1.5, 2) was observed by scanning electron microscopes (SEM, Hitachi TM4000Plus and FESEM, JEOL JSM-7900F) with an acceleration voltage of 5 kV. The morphology of Ti_3_C_2_T_x_ MXene was observed by transmission electron microscopy (TEM; JEM 2100 F) with an acceleration voltage of 200 kV. The surface properties were determined by an atomic force microscope (AFM, CSPM5500). The chemical components and structure over a range of 4000–400 cm^−1^ were measured by a Fourier-transform infrared spectroscopy spectrometer (FTIR, Nicolet iS10). The crystalline structures were characterized by wide-angle X-ray diffraction (XRD, Rigaku D/Max 2550) in the 2θ range from 3° to 50° with Cu Kα radiation (*λ* = 1.5418 Å). The scanning rate was 5° min^−1^. The chemical composition of the Ti_3_C_2_T_x_-based aerogel surfaces was examined by X-ray photoelectron spectroscopy (XPS, ESCALAB 250) with an X-ray source (Al Kα hυ = 1486.6 eV). The Raman spectra from 100 to 2000 cm^−1^ were analyzed using a 632 nm laser by a Raman confocal micro-spectrometer (LabRAM HR Evolution, Horiba, France). The specific surface area and pore size distribution were measured by nitrogen adsorption/desorption isotherms through Brunauer–Emmett–Teller (BET, Autosorb-iQ-C).

*Pressure-sensing performances:* The different pressure conditions were achieved by a digital force gauge (M5-5, Mark10), electrodynamic measuring table (ESM303, Mark10) and compression testing machine with TRAPEZIUM X pressure acquired software (AGS-X 500 N, Shimadzu, Japan). The real-time current change of the sensor was measured by connecting the digital multimeter (Keithley DMM7510) with 0.1 V DC voltage provided by a DC power supply (DPS-305BM). The response (R_I_) was defined as relative current change (ΔI/I_0_), where ΔI represents the change of the current between the loading pressure state and the initial state. Additionally, the sensitivity (S) of the pressure sensor was defined as δR_I_/Δp, where p refers to the intensity of pressure. The electrical hysteresis was defined as ratio of the area beneath ΔI/I_0_ curves under loading and unloading.

1 $$ Hysteresis \left( \% \right) = \frac{{S_{{{\text{unloading}}}} - S_{{{\text{loading}}}} }}{{S_{{{\text{loading}}}} }} \times 100\% $$

where S_loading_ and S_unloading_ represent the integral area of the ΔI/I_0_ and pressure curves under pressure loading and unloading, respectively.

### Finite Element Simulation of the Stress Distribution

The COMSOL Multiphysics software was utilized to conduct the finite element (FE) simulation. For simulating the stress distribution, the “Solid mechanics” module, coupled with the “Stationary” study, was employed. First, we build the geometric model of the pressure sensor. The laminated aerogel was set to be six rectangles with 4 mm width and 0.005 mm height, and the applied force was distributed on a semicircle given a non-deformable metal material with a radius of 1 mm. Moreover, six contact pair nodes were set between the stress loaded object and the aerogel top surface layer, the aerogel lamellae, respectively. Subsequently, the left and right boundaries of the aerogel lamellae were set as fixed constraint node and the mesh was constructed on the models by Free Triangular feature node. Ultimately, the computation was executed to acquire the distribution of the stress for aerogel when subjected to different pressures.

## Results and Discussion

### Preparation and Characterization of MX/CS/PVDF Aerogels

**Fig. 2:**
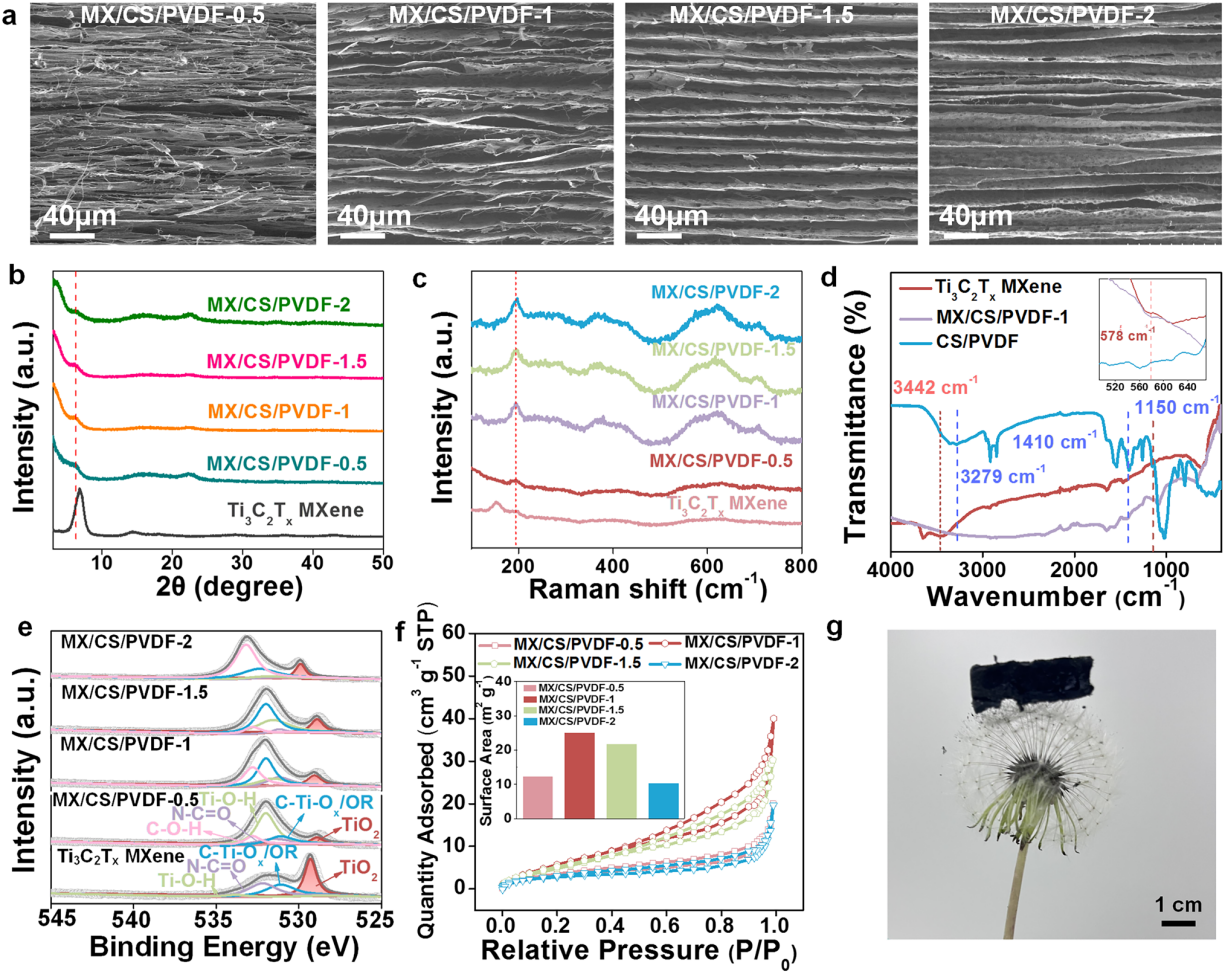
Characterization of MX/CS/PVDF aerogels. **a** SEM images of the cross-section morphologies. **b** XRD patterns and **c** Raman spectra of Ti_3_C_2_T_x_ nanosheet and MX/CS/PVDF aerogels. **d** FTIR spectra of Ti_3_C_2_T_x_ nanosheet, CS/PVDF, and MX/CS/PVDF-1 aerogel. **e** O 1*s* XPS spectra of Ti_3_C_2_T_x_ nanosheet, MX/CS/PVDF aerogels. **f** N_2_ adsorption–desorption isotherms of MX/CS/PVDF aerogels. **g** Photograph of an ultra-lightweight aerogel cut into 2 × 2 cm^2^ pieces placed on the taraxacum mongolicum

The fabrication process of Ti_3_C_2_T_x_ MXene/CS/PVDF (MX/CS/PVDF) composites aerogels is displayed in Fig. S1. The monolithic Ti_3_C_2_T_x_ (lattice stripe spacing: ~ 0.251 nm, Fig. S2), CS, and PVDF fibers (Fig. S3a) suspension were mixed uniformly followed by freeze-drying to yield an aerogel (Fig. S3b). SEM images of the interconnected and lamellar-structure aerogel (Fig. 2a) revealed the evolution of MX/CS/PVDF-x aerogel morphology with increased Ti_3_C_2_T_x_ concentrations. MX/CS/PVDF-0.5 showed a tighter structure with less space between the lamellae. The space became wider and the structure evolved more loosely in MX/CS/PVDF-1. However, higher Ti_3_C_2_T_x_ content resulted in the congregating and compact structure of the aerogel, because of the strong intermolecular forces between Ti_3_C_2_T_x_ nanosheets. SEM and elemental mapping images of MX/CS/PVDF lamellae indicated that Ti_3_C_2_T_x_ were evenly distributed in aerogel due to the strong hydrogen bond between CS and Ti_3_C_2_T_x_. The PVDF short fibers were randomly distributed within the aerogel, locating between the layers of the aerogel sheet, embedded within the aerogel lamellae, and on the surface of the aerogel lamellae (Figs. S4-S6). The AFM images proved the evolution of aerogel surface roughness and morphology with increased Ti_3_C_2_T_x_ concentrations (Fig. S7).

The structure of MX/CS/PVDF aerogels was characterized by XRD, Raman, FTIR, and XPS. The XRD pattern showed that the (002) peak shifted toward the low-angle direction and a decrease in intensity, indicating expanded layer spacing of the Ti_3_C_2_T_x_ layer caused by the addition of CS and PVDF nanofibers (Figs. 2b and S8). The A_1g_, relevant to the out-of-plane vibration of Ti and C atoms, shifted from 151 to 194 cm^−1^ and the cell surface unit deformed in Confocal Raman spectroscopy (Fig. 2c) [36]. The robust hydrogen bonding between CS and Ti_3_C_2_T_x_ was confirmed by broadening and blue shift of peaks around 2240 cm^−1^ of MX/CS/PVDF-1 compared to Ti_3_C_2_T_x_ and CS/PVDF aerogel (Fig. 2d) [37]. Moreover, the vibration peaks of Ti–O, -NH, -CF_2_, and β phase could be observed in the FTIR spectra, indicating the successful preparation of MX/CS/PVDF aerogel [38]. The chemical states and bonding configurations were revealed by XPS patterns (Fig. 2e). The O 1*s* peak showed that the oxidation of Ti_3_C_2_T_x_ was retard as a result of the formation of hydrogen bonds between Ti_3_C_2_T_x_, CS, and PVDF. The involvement of CS and PVDF reduced the peak area of the TiO_2_ phase. However, an excessive amount of Ti_3_C_2_T_x_ increased the oxidation process due to the exposure of susceptible oxidation groups [39].

The MX/CS/PVDF-x aerogel had a high specific surface area of 25.025 m^2^ g^−1^ (Fig. 2f inset) and ultralow density (ρ < 6.86 mg cm^−3^) of MX/CS/PVDF-1, which could be placed on the surface of the taraxacum mongolicum without deformation (Fig. 2g). The N_2_ adsorption/desorption isotherms of MX/CS/PVDF-x exhibited a representative H3 hysteresis loop and II isotherms (Fig. 2f), indicating that the adsorption of Ti_3_C_2_T_x_-based aerogel occurred as a multilayer reversible adsorption process on the surface of mesoporous or macroporous [40–42]. Moreover, the inflection point was located near the monolayer adsorption and the multilayer absorption was developed with P/P_0_ increasing. The limit equilibrium adsorption value cannot be observed from the isotherm due to the endless absorption layers.

The stable mechanical properties of MX/CS/PVDF were demonstrated by repeated compression–relaxation tests (Fig. S9a-c). Compared with Ti_3_C_2_T_x_ MXene/CS aerogel (Fig. S9d-f), the MX/CS/PVDF aerogel exhibited elasticity that could fully recover to the original state as the load pressure (35.39 -1769.5 kPa) was removed. The durability of MX/CS/PVDF-1 revealed almost no attenuation of the ultimate stress during 100 cycles of repeated compression–relaxation. In contrast to Ti_3_C_2_T_x_ MXene/CS, the addition of PVDF short fibers served as reinforced phase remarkably enhanced the durability and mechanical stability of aerogel. When suffering high pressure, the reinforced phase buffered and dispersed most of the stresses, prevented irreversible deformation and prevented damage to the conductive pathways of the substrate materials. The MX/CS/PVDF aerogel exhibited extraordinary performances such as lightweight and mechanical properties compared with conductive aerogel in previous work (Table 1).

**Table 1:** Comparison of the density and maximum stress between the prepared conductive aerogel in this article and previous articles

Density (mg cm^−3^)	Maximum stress (kPa)	Material	References
66.3	~ 89.4 (100 cycles)	MXene@carboxylated carbon tube/carboxymethyl chitosan	[57]
10.3	~ 58	Polyimide/carbon tube	[58]
25	~ 49.8	MXene/aramid nanofibers	[59]
10	~ 1.2	Polysiloxane cross-linked MXene	[60]
50	~ 40	Sodium alginate/MXene/polydimethylsiloxane	[61]
17		Graphene oxide/dopamine/polyaniline	[62]
7–20.7	~ 30	MXene/chitosan	[63]
14.67		Polypyrrole/cellulose acetate	[64]
13.3	~ 30	Alkali lignin/carbon nanofiber	[65]
11.2		Poly(3,4-ethylenedioxythiophene):polystyrene sulfonic acid/carbon nanofiber	[66]
6.7		Carbon nanofiber-Graphene oxide/glucose-kaolin carbon	[67]
25.6	~ 22.5 (3000 cycles)	R-graphene oxide/polyimide	[68]
8.16	~ 75 (300 cycles)	Graphene oxide/hydroxypropyl methyl cellulose	[69]
20.54	~ 800 (100 cycles)	Polyacrylonitrile nanofibers/polyvinyl alcohol/carbon tube/hydrophobic octadecylamine functionalized r-graphene oxide	[70]
12.6–26.5		FeS_2_/carbon tube	[71]
7.48	~ 6 (100 cycles)	Carbon nanofiber/carbon tube/MXene	[72]
12		AgNWAs/carboxymethyl cellulose	[73]
42.7	~ 50	Chitosan/carbon tube	[74]
260		MXene/carbon nanofiber/thermoplastic urethane	[75]
200–300		Carbon tube	[76]
228	~ 800 (1000 cycles)	Graphene armor	[77]
6.86	~ 108 (100 cycles)/1769.5 (1 cycle)	Ti_3_C_2_T_x_ MXene/chitosan/polyvinylidene difluoride	This work

### Pressure-Sensing Performance

**Fig. 3:**
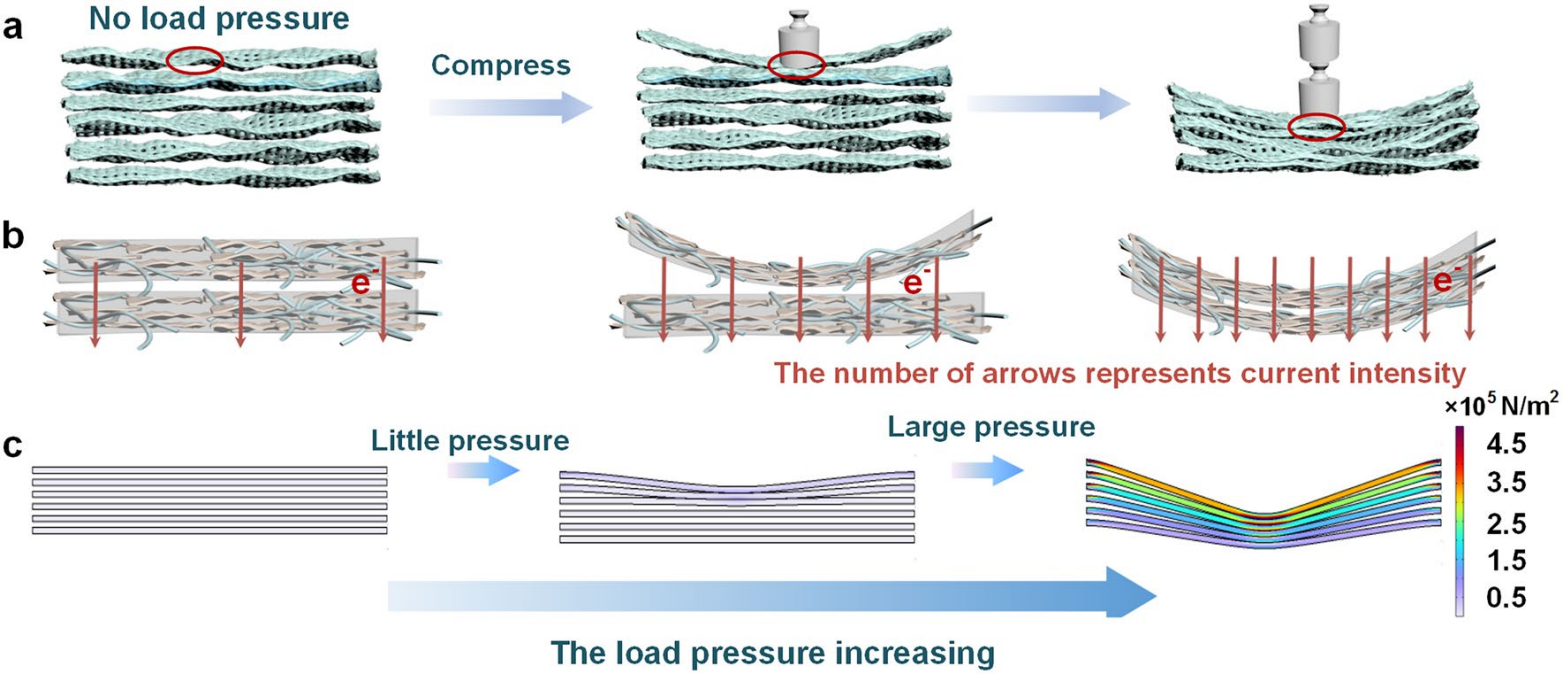
Piezoresistive effect of MX/CS/PVDF aerogel pressure sensor. **a** Schematic of the pressure sensor under different load pressures. **b** Illustration of MX/CS/PVDF lamellae deformation states and the electron transport process under different pressures. **c** Compressing deformation mechanism of MX/CS/PVDF-1 pressure sensor explained by FE simulation. The color contours represent the microscopic strain of the aerogel structure during the deformation process

The pressure sensor was fabricated by fixing the copper wires as electrodes on the top and bottom surfaces of the MX/CS/PVDF aerogel and then simply encapsulated with waterproof and flexible polyurethane (PU) with 14 μm thickness (Fig. S10). The effect of the encapsulation layer thickness and sensitive materials thickness on the sensing performance was investigated in Supporting Information (Figs. S11-S14). Figure 3a demonstrates the strain behavior where the top thin lamellae of MX/CS/PVDF were bent and deformed under ultralow applied pressure, while the inner Ti_3_C_2_T_x_ nanosheets become closer. When the load pressure increased, the top thin lamellae and the bottom of MX/CS/PVDF underwent deformations. Therefore, the conductive pathway of MX/CS/PVDF aerogel increased with an accelerated electron transport rate, resulting in a larger relative current (Fig. 3b). The sharp increase in conductive pathways was contributed by the deformations of Ti_3_C_2_T_x_-based lamellae and the increase in contact area of MX/CS/PVDF lamellae. As the load pressure continued to increase, MX/CS/PVDF lamellae stopped deforming. The contact area between the lamellae expanded until full contact was established and conducting pathways were no longer increased, while the electron transport rate reached maximum.

To illustrate the working mechanism of the MX/CS/PVDF aerogel-based pressure sensor, finite element simulations were conducted to calculate the stress distribution during the loading process (Fig. 3c). By applying a small load pressure, the stress was distributed throughout the top aerogel lamellae, causing a fraction of the aerogel to deform when it came into contact with one another. The stress distribution range and the contact area between the aerogel lamellae both grew with increasing load pressure. This process of change aligned with the actual situation.

**Fig. 4:**
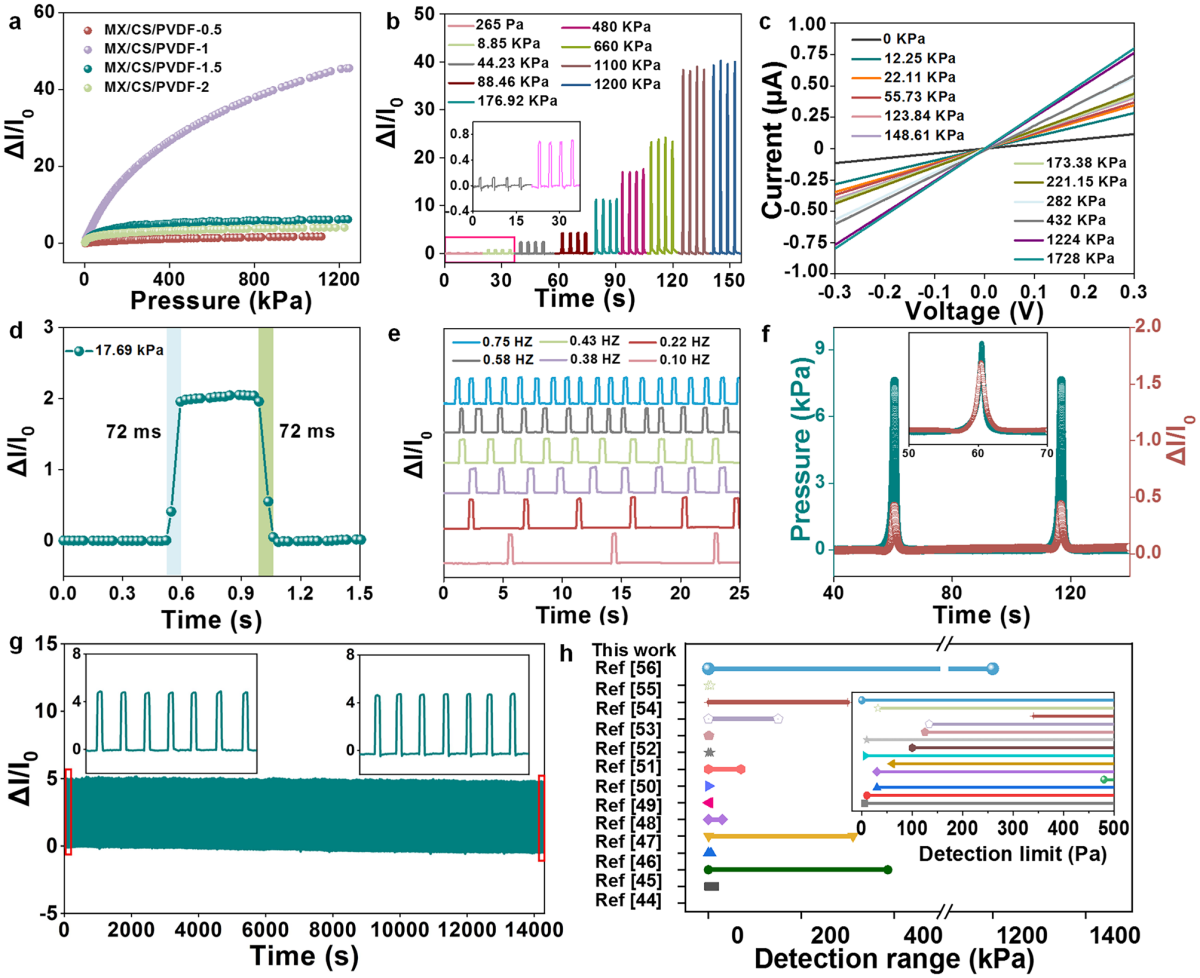
Pressure-sensing performances of MX/CS/PVDF-1 aerogel pressure sensor. **a** Response-pressure curves of MX/CS/PVDF with different Ti_3_C_2_T_x_ concentrations. **b** Relative current changes under load pressure of 265 Pa-1200 kPa. **c** The I-V curves of the MX/CS/PVDF-1 sensor under different load pressures. **d** Response and recovery time at a pressure of 17.69 kPa. **e** The variation of I-t curves with different running frequencies load pressure. **f** Instantaneous response of I-t and P–t curves. **g** Durability test under 6000 loading/unloading cycles at a pressure of 22.12 kPa, inset shows the enlarged curve for the first seven cycles and last seven cycles. **h** Performance comparison of piezoresistive sensors

The sensing performances of MX/CS/PVDF aerogel were measured under various levels of pressure. MX/CS/PVDF-1 exhibited the highest sensitivity in the pressure range of 0–1200 kPa **(**Fig. 4a). The mechanism was that the sensitivity of the pressure sensor was primarily associated with the number of conductive pathways inside the aerogel before and after pressure loading and unloading, and the number of conductive pathways of the MX/CS/PVDF-x sensor was determined by the content of Ti_3_C_2_T_x_ nanosheets and specific surface area. Owing to low Ti_3_C_2_T_x_ content and specific surface area, a sufficient number of conductive pathways could not be established inside MX/CS/PVDF-0.5 aerogel, and the conductive pathways did not change much even after loading pressure (Fig. S15a). For MX/CS/PVDF-1.5 and MX/CS/PVDF-2, due to the excessively high Ti_3_C_2_T_x_ content and the reduced specific surface area, sufficient conductive pathways were already established within the aerogel without pressure loading being received, so that the number of conductive pathways did not change much when pressure loading was applied (Fig. S15c, d).

With load pressure continuously increasing from 265 Pa to 1200 kPa, the relative current intensity gradually increased, allowing for effectively distinguishing different levels of load pressure (Figs. 4b and S16). The sensitivity ratio for MX/CS/PVDF-1 was 0.11 kPa^−1^ in the range of 0–106 kPa (Fig. S15b). As applied pressure increased from 106 to 400 kPa, the sensitivity decreased to 0.055 kPa^−1^, and when the pressure was higher than 400 kPa, the sensitivity was decreased to 0.022 kPa^−1^. Therefore, the sensing performance of MX/CS/PVDF-1 with the optimal output signal would be explored under different input load pressures. Figure 4c illustrates the excellent linear relation of the current–voltage (*I-V*) curves from -0.3 to 0.3 V under various load pressures from 0 to 1728 kPa. It indicated that the ohmic contacts were formed between the MX/CS/PVDF-1 sensing layer and Cu electrodes [43]. Notably, as the load pressure increased, the slope of the *I-V* curves became steepen, indicating a decrease in device resistance and a subsequent rise in current.

This MX/CS/PVDF-1-based pressure sensor exhibited a rapid response/recovery time of 72 ms under 17.69 kPa load pressure (Fig. 4d). MX/CS/PVDF-1 emerged with extraordinary stability and distinguishable response signal at different frequencies from 0.1 to 0.75 Hz (Fig. 4e), from 0.54 Hz to 1.96 and 2.47 Hz (Fig. S17). The low hysteresis of 13.69% is demonstrated in Fig. S18 and further confirmed by the output current change signal and the consistency of the input load pressure and current signals during pressure loading and unloading (Fig. 4f). Additionally, little attenuation and a nearly identical relative current change were observed during 6000 cycle tests at 22.12 kPa pressure. The identical current amplitude of the initial and end states proved the durability (Fig. 4g). The long-term stability of the MX/CS/PVDF-1 pressure sensor was confirmed by the consistency of the initial current and the relative current change under 15.91 kPa over a period of one month (Fig. S19). The initial current was stable for 31 days and the relative current response to pressure had no significant attenuation. MX/CS/PVDF-1 pressure sensors demonstrated superior stability under varying ambient humidity and temperature, and it was discussed extensively in Supporting Information (Fig. S20).

The pressure sensor can detect a low load pressure of 6.25 Pa (Fig. S21). It attributed to an ultralow compression modulus of 0.234 kPa of MX/CS/PVDF-1 that deformed under ultralow load stress and generated visual electrical signals (Fig. S12a). There was a factor responsible for this instance that the elongated chain structure was formed in aerogel by hydrogen bonding between chitosan and Ti_3_C_2_T_x_ for the increased freedom degree of molecular movement. Moreover, the pressure sensor demonstrated a revisable deformation under high pressure due to the introduction of PVDF short fibers which ensured the reversible output response signals under wide detection range (Fig. S22a-c). Taken together, the detection ranges of the MX/CS/PVDF-1 outperformed previously reported aerogel-based pressure sensors, with a low detection limit of 6.25 Pa, maximum detection range of 1200 kPa, and excellent stability (Fig. 4h) [44–56]. The effect of PVDF fibers length and the laminated structure or random structure on the sensing performance was further investigated in Supporting Information (Figs. S22-S24).

**Fig. 5:**
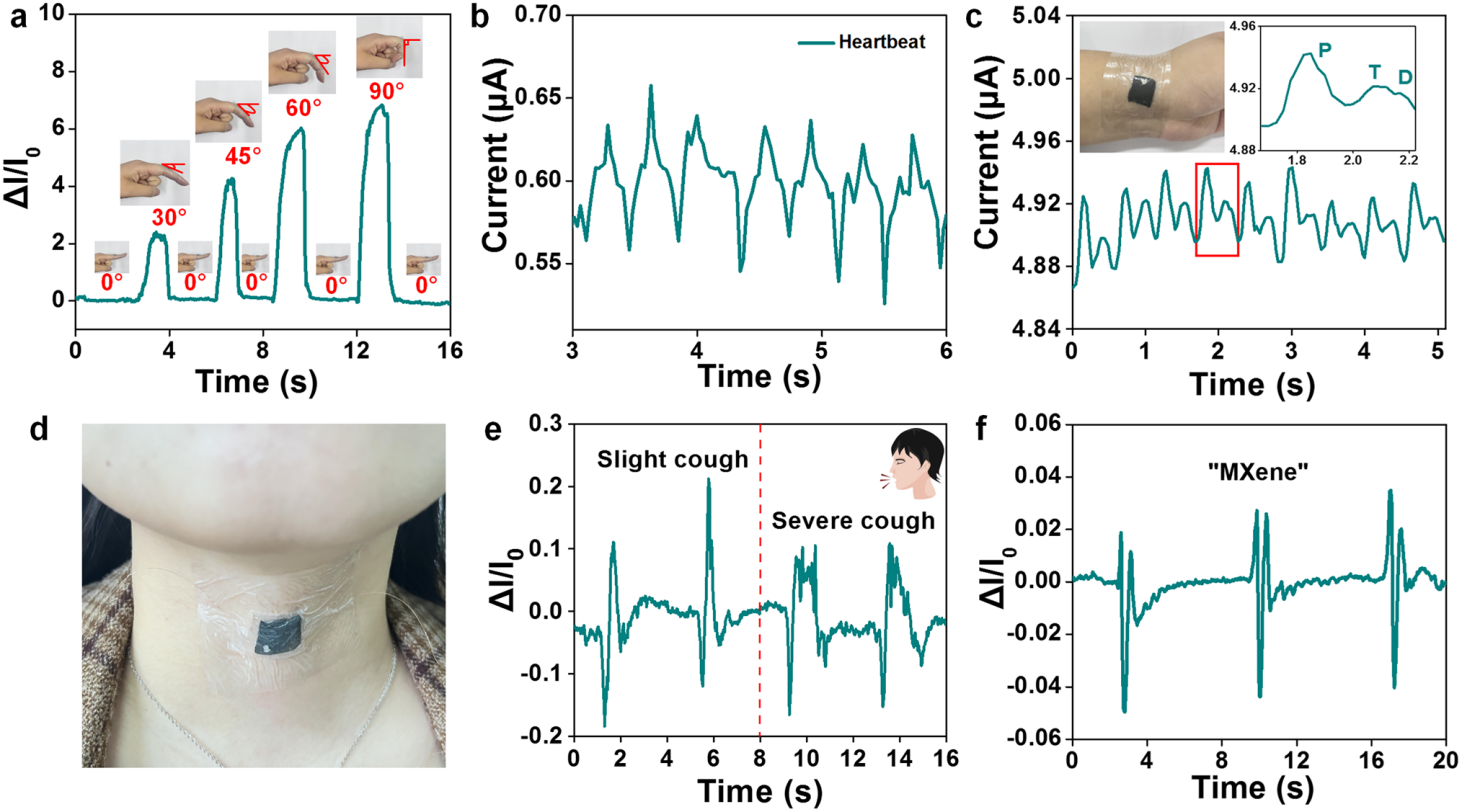
Real-time monitoring of human physiological signals using the piezoresistive sensor. **a** Relative current change of finger bending angle from 0 to 30, 45, 60, 90 degrees. **b** Real-time recording of the heartbeat waveform. **c** Real-time recording of the wrist pulse waveform (The inset illustrates the enlarged view of the pulse vibration waveform). **d** Detection of human physiological signals when the MX/CS/PVDF-1 was fixed on the throat of a 24-years-old volunteer: **e** Cough, and **f** Say “MXene”

To further evaluate the practical feasibility, a MX/CS/PVDF-1 pressure sensor was placed on the joints and vocal cords of the human body to detect human physiological signals. It was observed that the action and physiological state of the human body could be distinguished by comparing the output signal shape and intensity of the curves in Fig. 5. The pressure sensor can effectively distinguish items weighing from 100 mg (22.6 Pa) to 20 g (1.48 kPa) and bending angles in the range from 0° to 90° (Figs. S25a and 5a). The output signal intensity varied significantly when the volunteers pressed manually between the relative low-pressure range and high-pressure range (Fig. S25b). The heartbeat and the pulse, as an important physiological signals of the human body, was detected by attaching to the chest and wrist of a 24-year-old volunteer (Fig. 5b, c). The periodic and stable wrist pulse with a regular beating of 108 beats per minute was illustrated, where three faint characteristic peaks percussion wave (P), tidal wave (T), and diastolic wave (D) of the human pulse could be clearly distinguished (Fig. 5c). Furthermore, human actions including swallowing, yawning, and coughing could be precisely detected when the sensor was placed on the throat (Figs. 5c, e, and S25c, d). Meanwhile, it can accurately capture the throat vibration during human pronunciation, which showed significant differences in waveforms of “Wearable,” “Sensor,” and “MXene” (Fig. 5f and S25e, f).

### Dialect Speech Recognition Based on MX/CS/PVDF-1 Pressure Sensor

For non-standard language people, it is extremely complicated to communicate with the natives using standard language when they need to hospitalize out-of-town or migrant work. The same dialect shares similar spoken speed, syllables, and pitches, and therefore, generates specific electrical signal waveforms, which can be effectively distinguished between dialects. When saying the same word in different dialects, there are similarities in both syllables and pronunciation patterns for the same language (Fig. S26). The MX/CS/PVDF-1-based pressure sensor demonstrated low detection limit (6.25 Pa), wide detection range (~ 1200 kPa), and low hysteresis (13.69%) that superior the previous pressure sensor for speech recognition and could acquire stable signals (Table S1).

**Fig. 6:**
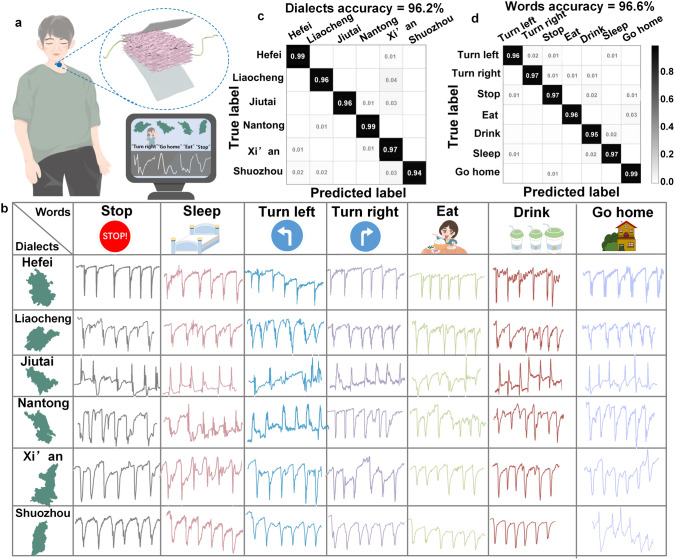
Dialect speech recognition assisted by deep learning. **a** Pressure sensor was fixed on the throat for speech recognition. **b** Continuous current signal images generated by seven vocabularies in six Chinese dialects. **c** Confusion matrix of the CNN algorithm for six different Chinese dialects. **d** Confusion matrix of the CNN algorithm for seven words in different Chinese dialects

To satisfy the demand for dialect speech recognition, the MX/CS/PVDF-1 based pressure sensor was fixed on the throat of six dialect speakers from Nantong, Liaocheng, Jiutai, Shuozhou, Xi’an, and Hefei. The selected dialects were representative and were primarily associated with geographic distribution from each of the six provinces in the eastern, northern, central, western, and southern parts of mainland China. To further improve the accuracy of identification, the dialects with similar pronunciation such as Hefei and Nantong dialects belonging to the Mandarin of Jianghuai, Shuozhou, and Xi'an dialects belonging to the Central Plains Mandarin were selected. Moreover, the sensing signals were obtained for further training and recognition when the dialect speakers spoke seven daily instructional words such as “stop,” “sleep,” “turn left,” “turn right,” “eat,” “drink,” and “go home.” Deep learning algorithms are employed for dialect speech recognition to achieve precise categorization and dialect recognition (Fig. 6a). Convolutional neural networks (CNNs), a translation invariant classification algorithm based on sophisticated mathematical theory, are widely utilized to mimic the cognitive learning ability of the human brain for image processing and categorization. Here, we developed a CNN algorithm with an 11-layer network for accurate classification of dialect types and the common vocabulary in the different dialects (Fig. S27). Over 6888 and 4158 vocalization vibration signals on the throat were collected by the sensor for training a CNN model to identify 7 vocabularies and 6 Chinese dialects, respectively (Fig. 6b).

The filtered processed electric signals are presented to the input layer and then perform feature extraction across four convolutional layers. The model can be partitioned into four parts, and each portion contains at least one convolutional layer and one maximum pooling layer (MP). The effect of MP is to simplify the complexity, compress the eigenvalues, and reduce the computational effort. Additionally, the convolutional layer is followed by a batch normalization layer for reducing the covariance drift in the model and improving the stability of the model. Three fully connected layers utilized for the final prediction follow the four parts. It should be noted that the dropout layer in the fully connected layer serves to reduce overfitting by ignoring half of the hidden nodes. The activation function used after MP is the ReLU.

The deep learning model based on the CNN algorithm was split into two processes. The first process was model training, where the image size was set as 64 × 64 and the batch size was 512. The loss value started to converge after 6 epochs, and the accuracy of the training set reached 100% after 16 loss values. Eventually, the accuracy of the test set reached 96.2% and 96.6% for six Chinese dialects and seven words in different dialects (Fig. 6c, d).

## Conclusion

Here, we have fabricated MX/CS/PVDF-based pressure sensors with ultralight density and remarkable durability for dialect recognition. The elaborately designed MX/CS/PVDF-1-based pressure sensor exhibited rapid response/recovery time (< 72 ms) and a low detection limit (6.25 Pa), allowing for the detection of slight vibrations in the throat. During the process of dialectal speech recognition, over 6888 and 4158 vocalization vibration signals on the throat were obtained by saying 7 vocabularies and 6 Chinese dialects for training a CNN model. The recognition accuracy of dialect pronunciation information was 96.6% and 96.2%, respectively. This high-performance pressure sensor can have a significant role in human–machine interaction and health monitoring in the future to express instructions and acquire physiological information.

## Supplementary Information

Below is the link to the electronic supplementary material.

Supplementary file1 (DOCX 19968 KB)
