# Supplementary material for: Ti3C2Tx Composite Aerogels Enable Pressure Sensors for Dialect Speech Recognition Assisted by Deep Learning
Source: Nanomicro Lett. 2024 Dec 30;17:101. doi: 10.1007/s40820-024-01605-z (PMC11683042; doi:10.1007/s40820-024-01605-z)
Supplement: Supplementary file 1 — Supplementary file1 (DOCX 19968 KB) [file 40820_2024_1605_MOESM1_ESM.docx]

Supporting Information for

**Ti_3_C_2_T_x_ Composite Aerogels Enable Pressure Sensors for Dialect Speech Recognition Assisted by Deep-Learning**

Yanan Xiao^1,#^, He Li^1,#^, Tianyi Gu^1^, Xiaoteng Jia^1,^*, Shixiang Sun^1^, Yong Liu^1^, Bin Wang^1^, He Tian^2^, Peng Sun^1,3^, Fangmeng Liu^1,3,^*, Geyu Lu^1,3^

^1^ State Key Laboratory of Integrated Optoelectronics, College of Electronic Science and Engineering, Jilin University, Changchun 130012, P. R. China

^2^ School of Integrated Circuits, Tsinghua University, Beijing 100084, P. R. China

^3^ International Center of Future Science, Jilin University, Changchun 130012, P. R. China

^#^ Yanan Xiao and He Li contributed equally to this work.

*Corresponding authors. E-mail: [xtjia@jlu.edu.cn](mailto:xtjia@jlu.edu.cn) (Xiaoteng Jia); [liufangmeng@jlu.edu.cn](mailto:liufangmeng@jlu.edu.cn) (Fangmeng Liu)

**Supplementary Figures and Tables**


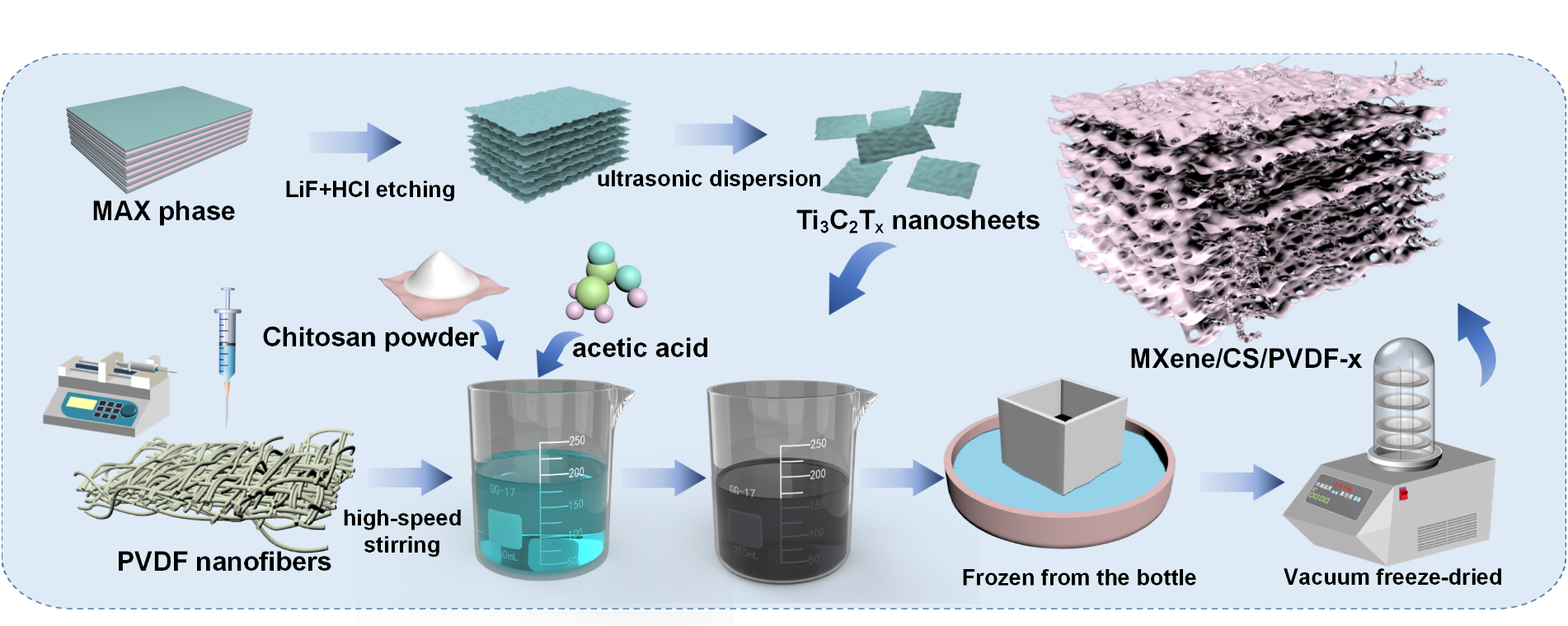


**Fig. S1** Schematic diagram of the fabrication process of MX/CS/PVDF aerogel

**
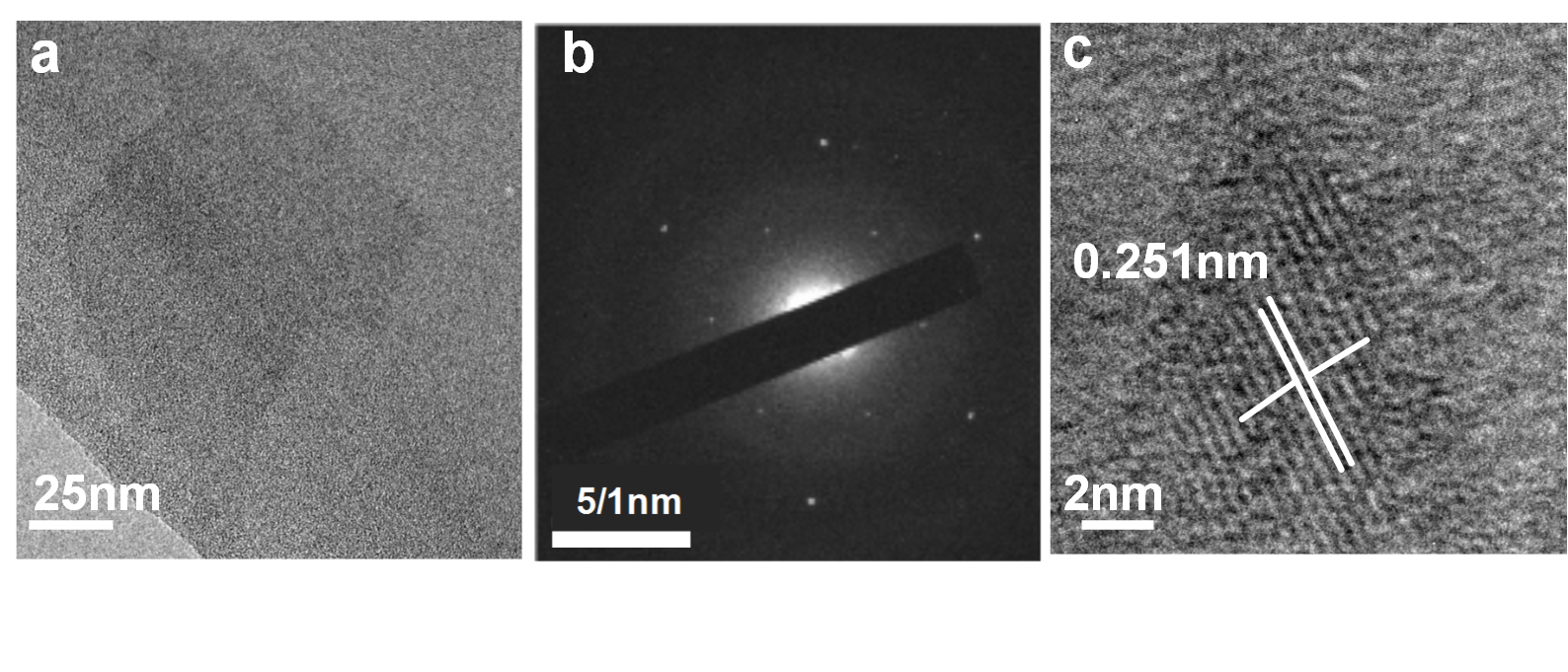
**

**Fig. S2** **a** TEM images of Ti_3_C_2_T_x_ nanosheet. **b** SAED pattern of Ti_3_C_2_T_x_ nanosheet. **c** HETEM image of Ti_3_C_2_T_x_ nanosheet

**
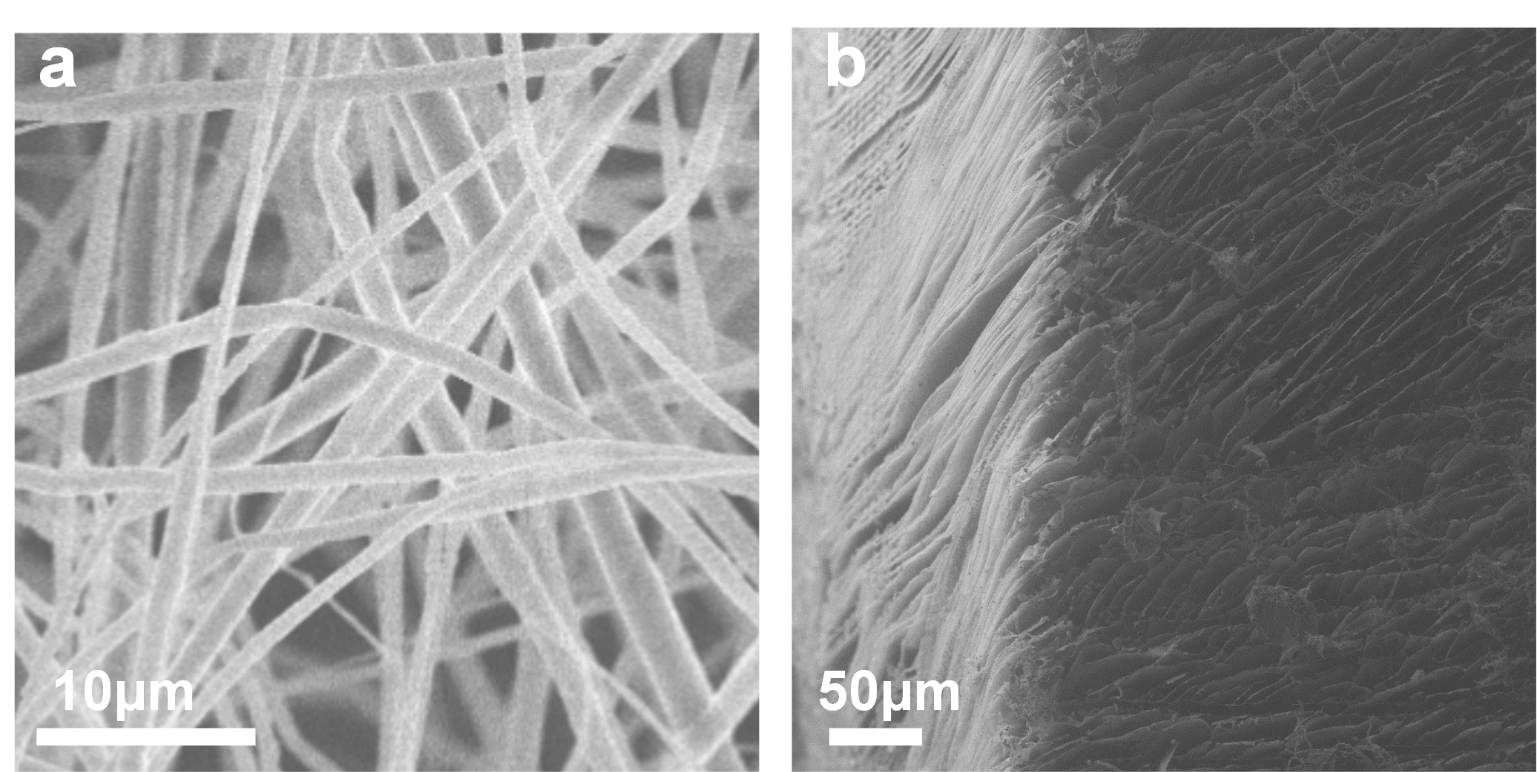
**

**Fig. S3** SEM images: **a** PVDF nanofibers and **b** microstructure of MX/CS/PVDF-1


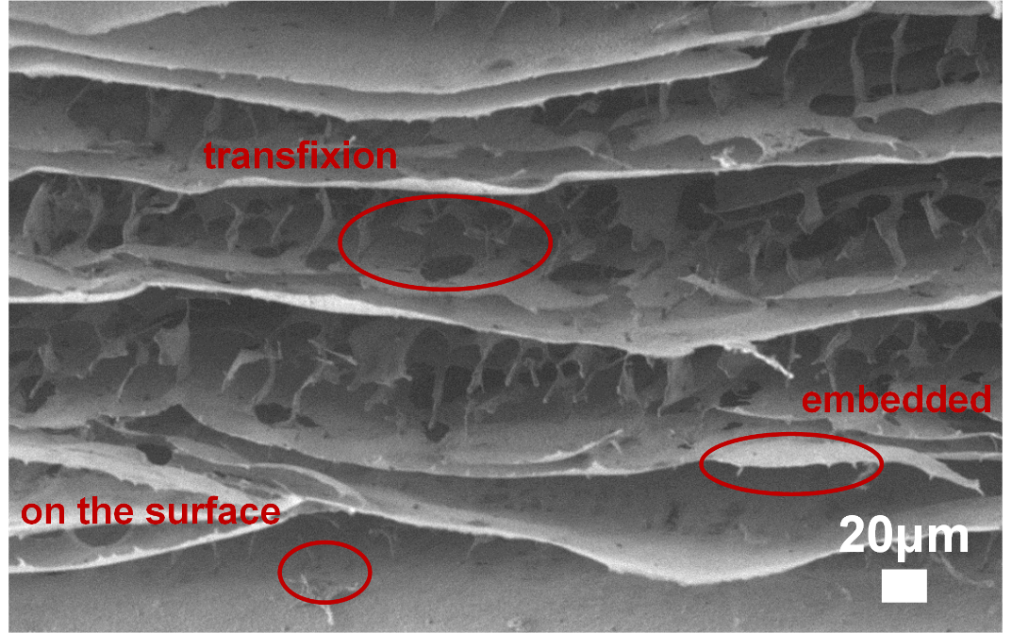


**Fig. S4** SEM images of the distributions of PVDF fibers in MX/CS/PVDF-1 aerogel

**
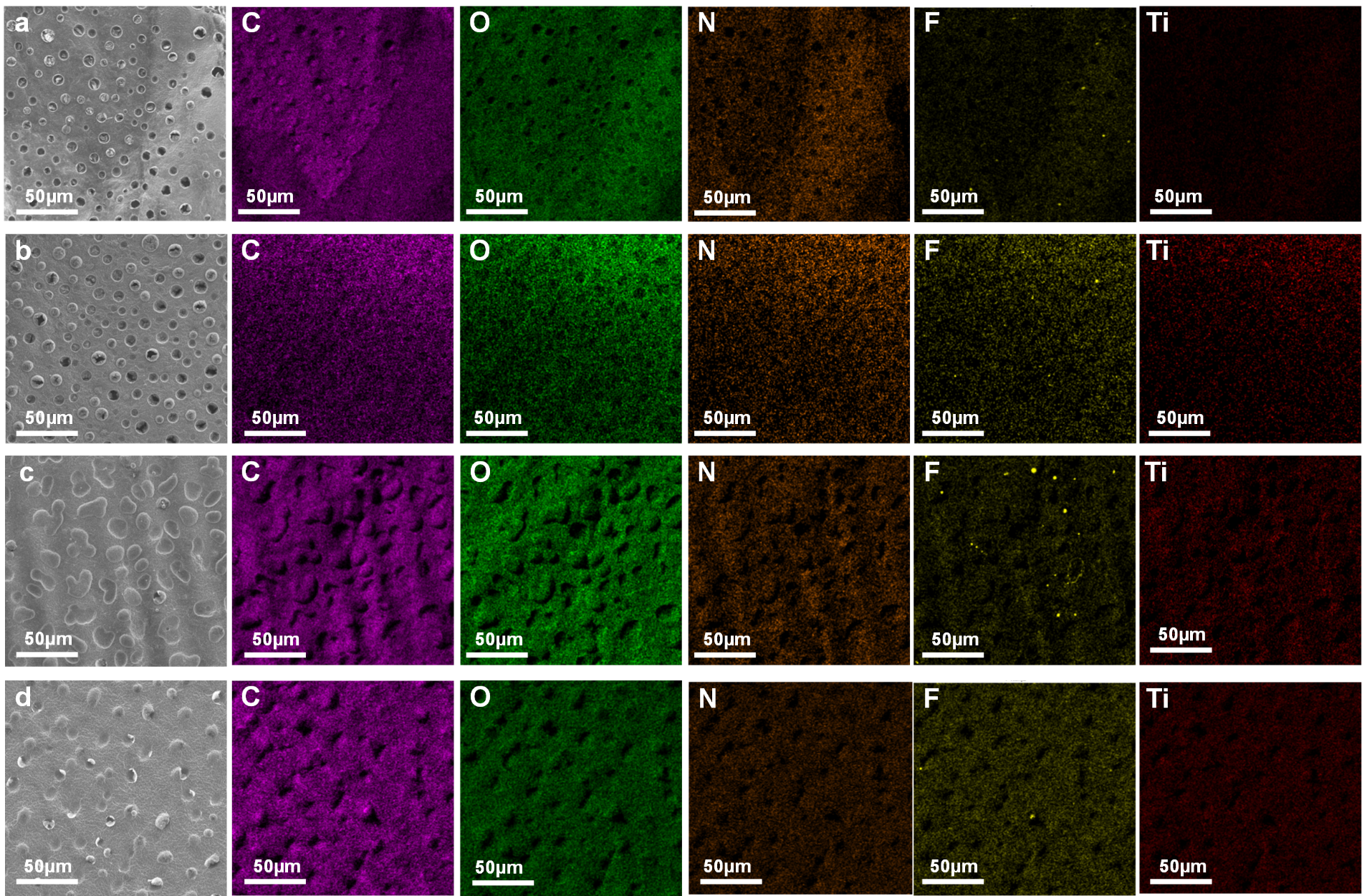
**

**Fig. S5** SEM and corresponding EDX elemental mapping images of **a** MX/CS/PVDF-0.5, **b** MX/CS/PVDF-1, **c** MX/CS/PVDF-1.5 and **d** MX/CS/PVDF-2

**
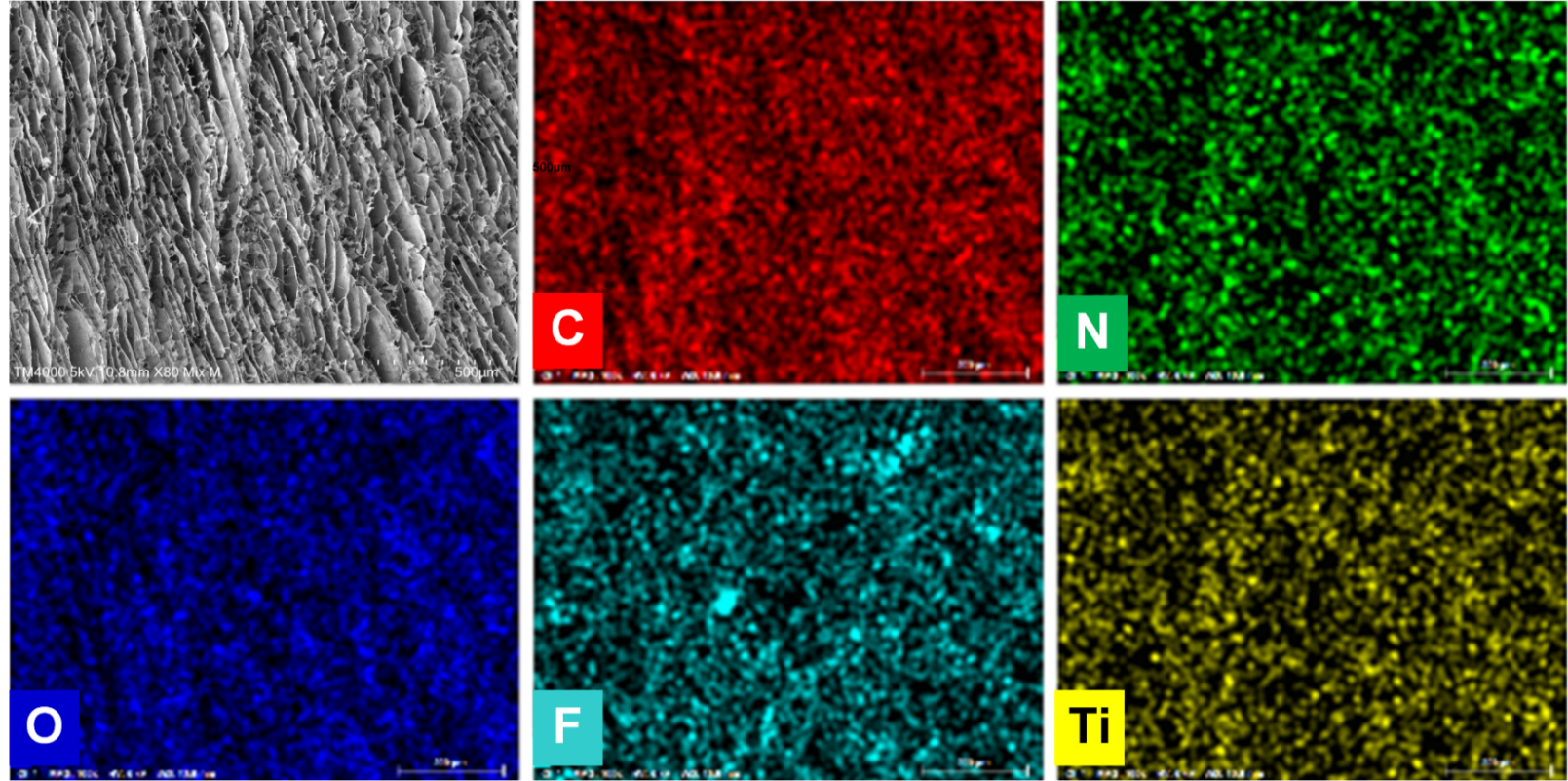
**

**Fig. S6** SEM and corresponding EDX elemental mapping images of MX/CS/PVDF-1

**
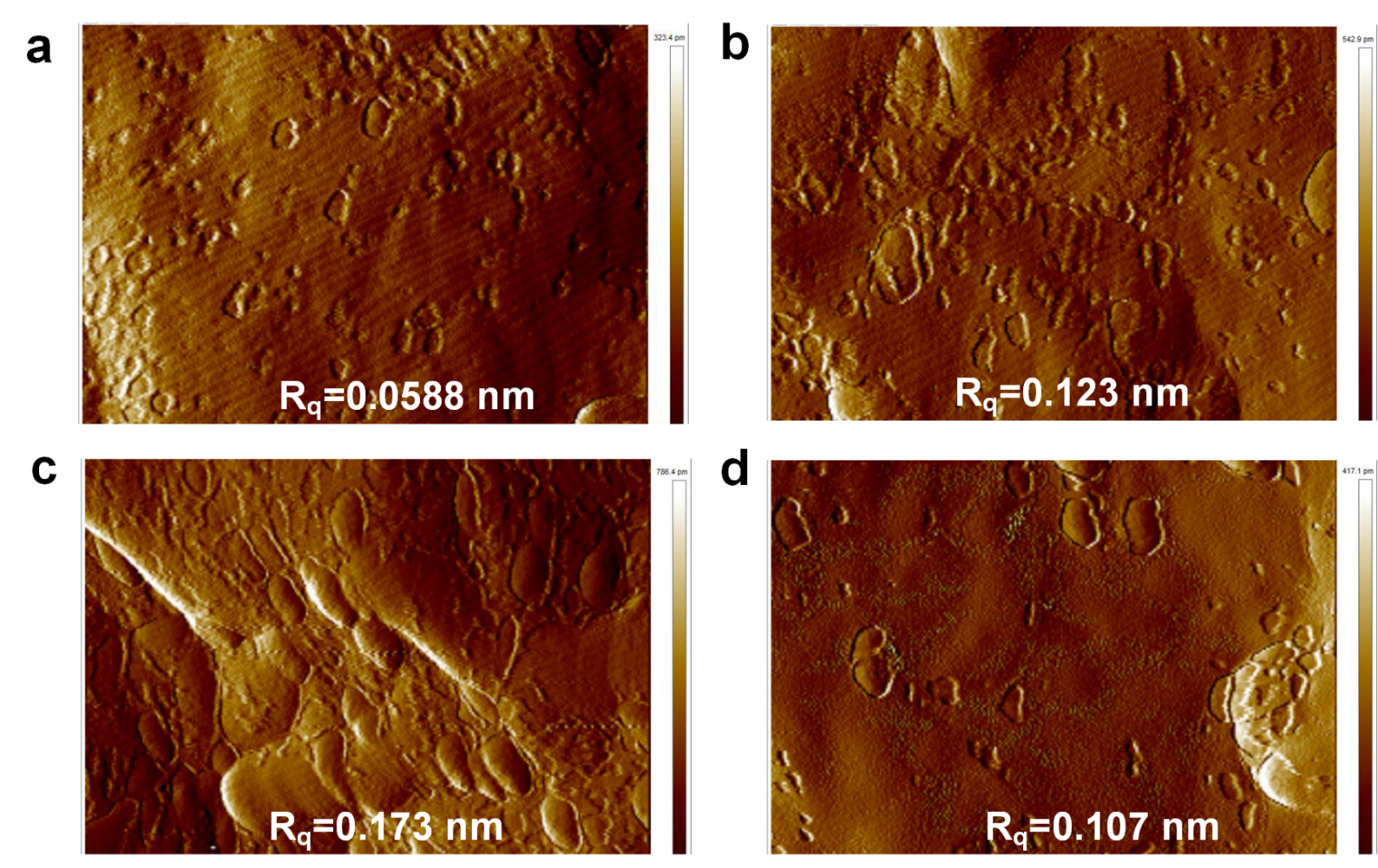
**

**Fig. S7** AFM images of **a** MX/CS/PVDF-0.5, **b** MX/CS/PVDF-1, **c** MX/CS/PVDF-1.5 and **d** MX/CS/PVDF-2

**
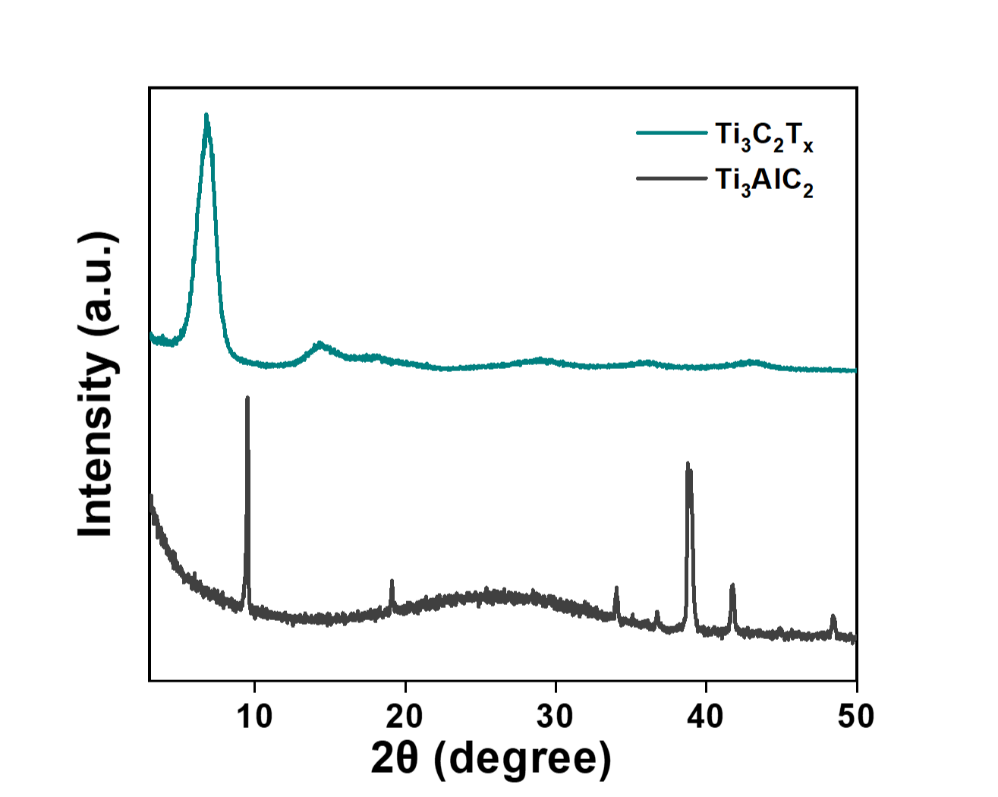
**

**Fig. S8** XRD spectra of single-layer Ti_3_C_2_T_x_ and precursors Ti_3_AlC_2_


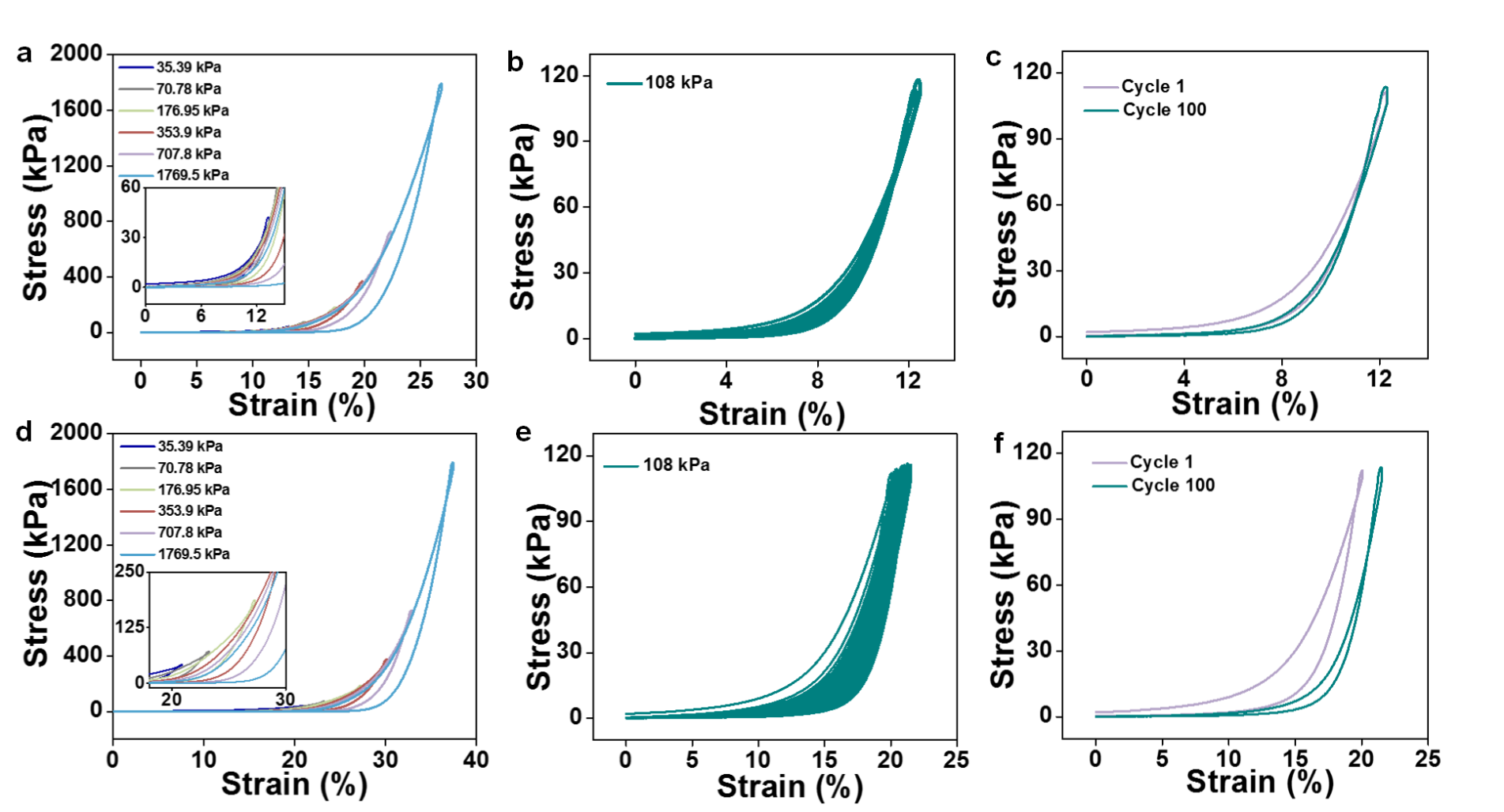


**Fig. S9** Stress-strain cycling curves under different pressure loads of **a** MX/CS/PVDF-1 and **d** Ti_3_C_2_T_x_ MXene/CS. Stress-strain curves at 100 cycles of **b** MX/CS/PVDF-1 and **e** Ti_3_C_2_T_x_ MXene/CS. Stress-strain cycle curves of 1^st^ and 100^th^ of **c** MX/CS/PVDF-1 and **f** Ti_3_C_2_T_x_ MXene/CS


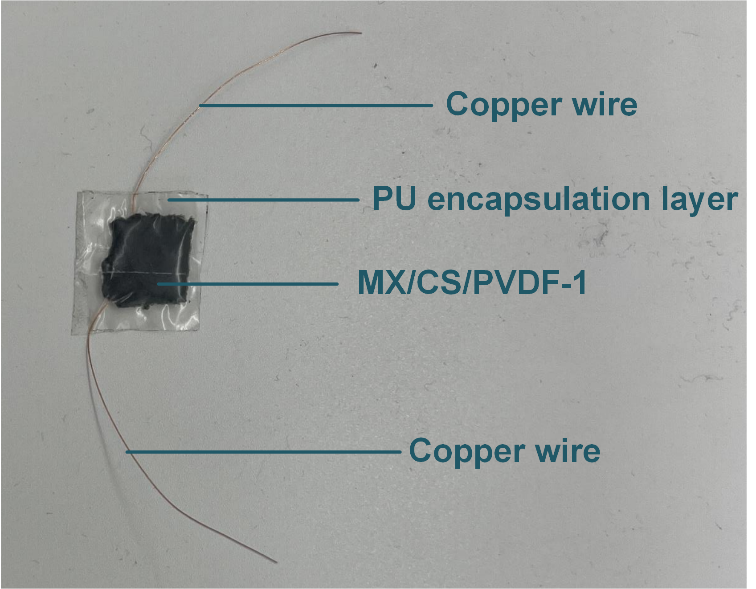


**Fig. S10** Photographs of MX/CS/PVDF-1 pressure sensor

**The effect of thickness of PU encapsulation layer on the sensitivity:** To investigate the effect of the thickness of the encapsulation layer on the detection sensitivity of the device, we encapsulated the top and bottom layers of the aerogel with one-layer PU (14 μm), two-layers PU (28 μm), and three-layers PU tape (42 μm), and tested the detection sensitivity of the three sensors.

As shown in Fig. S11b, there was no significant shift of sensitivity for MX/CS/PVDF-1 based pressure sensor with 14 μm and 28 μm PU encapsulation layer. When the encapsulation layer was 42 μm, the detection sensitivity was increased. However, the compression modulus of pressure sensors was increased and the detection limit also was increased with the thickness of encapsulation layer increasing (Fig. S12 and S13).


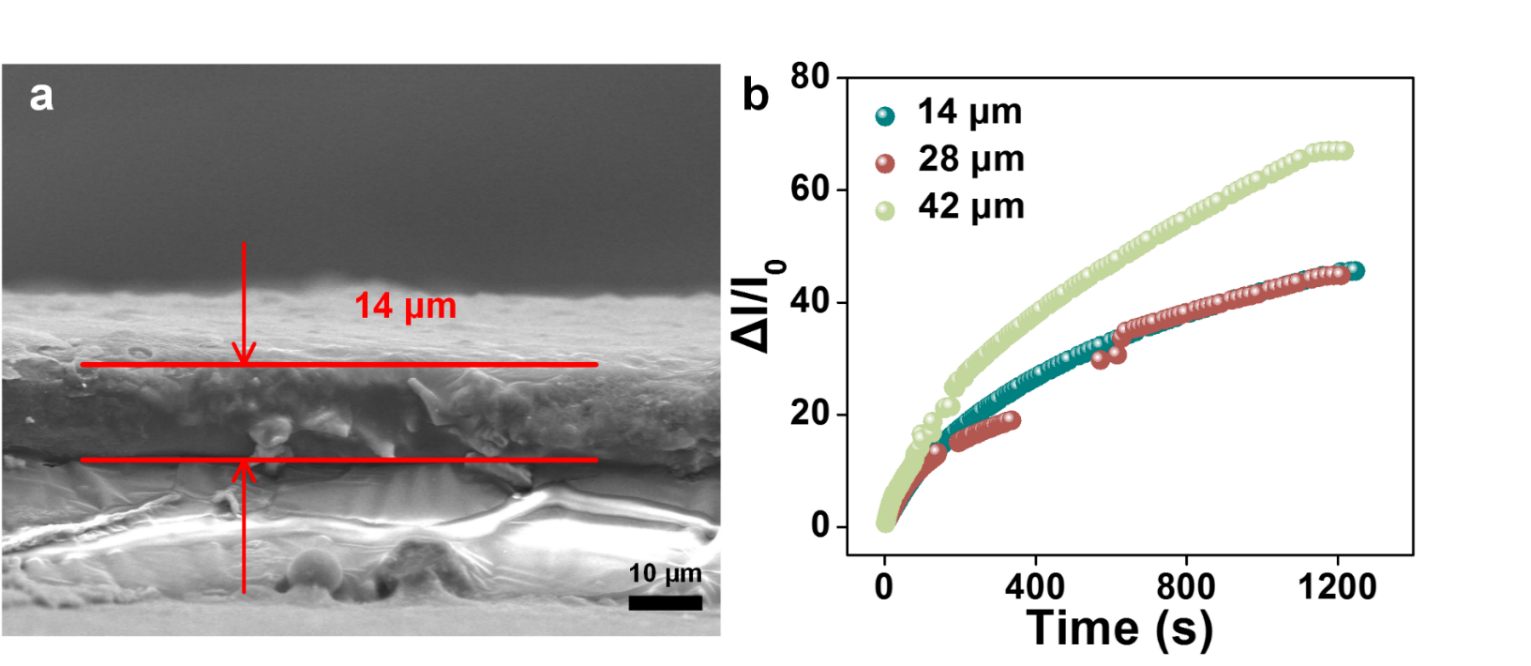


**Fig. S11** **a** The SEM image of cross-section of commercial PU tape. **b** The response-pressure curves of MX/CS/PVDF-1-based pressure sensor with different thickness PU encapsulation layer


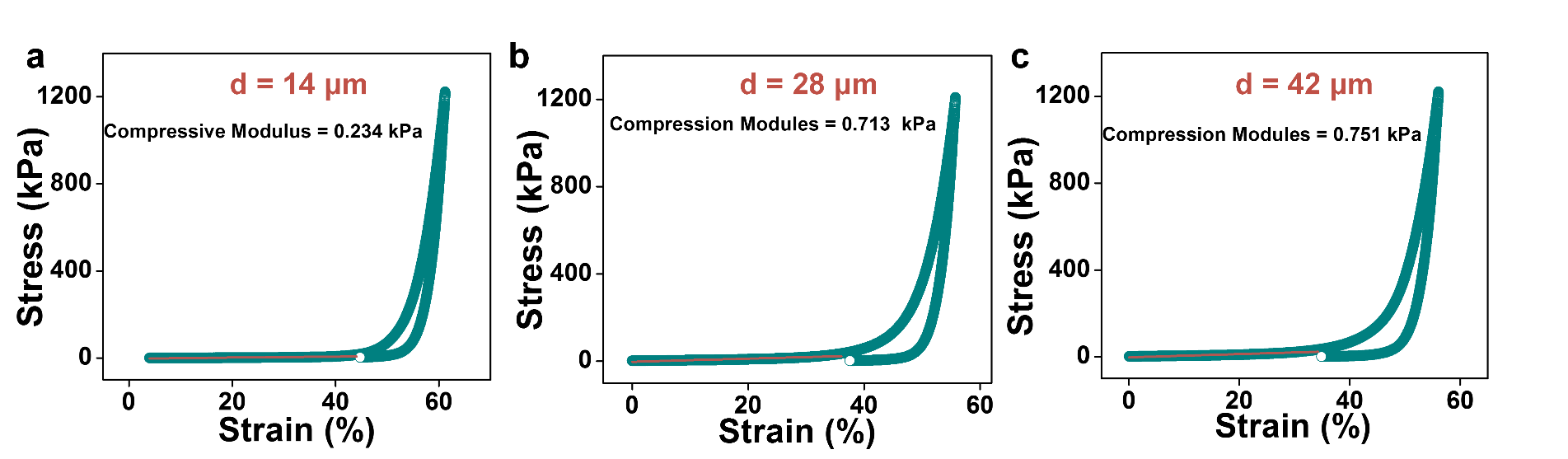


**Fig. S12** The compression modulus of MX/CS/PVDF-1 with different thickness PU encapsulation layer of **a** 14 μm **b** 28 μm and **c** 42 μm


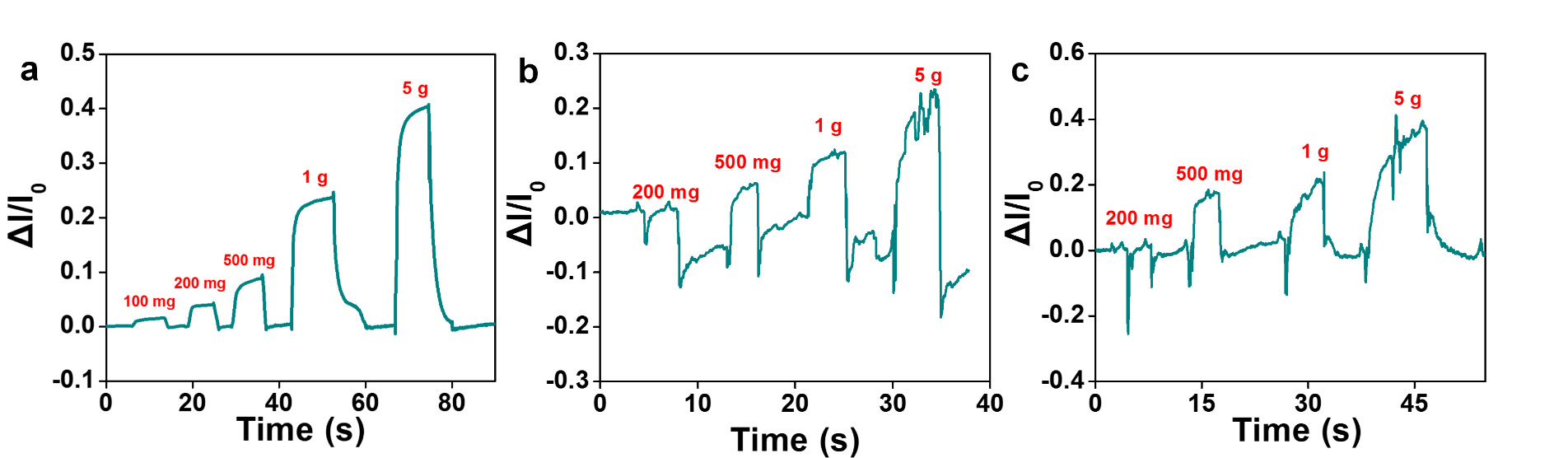


**Fig. S13** The sensing performances of MX/CS/PVDF-1 pressure sensor with different thickness PU encapsulation layer of **a** 14 μm **b** 28 μm and **c** 42 μm

**The effect of thickness of the sensitive layer on the sensitivity and durability:** MX/CS/PVDF-1 with different thicknesses of 0.25 cm, 0.5 cm, and 0.75 cm was fabricated by freeze-drying that 4 mL, 8 mL and 12 mL of MXene/CS/PVDF precursor was injected into 4×4 cm PTFE molds, respectively.

As demonstrated in Fig. S14a, compared with the pressure sensor with 0.5 cm, that with 0.25 cm and 0.75 cm exhibited a lower response. The sensing range of the pressure sensor with 0.25 cm was smaller than other thickness pressure sensors and the sensitivity was 0.23 kPa^-1^ in the range of 0-6.78 kPa (Fig. S14b), which was higher than the two others. However, the durability of 0.25 cm thickness was worse than other thickness pressure sensors. As shown in Fig. S14d, the baseline pressure sensor with 0.25 cm drifted dramatically during the pressure loading and unloading cycles. The baseline of that with 0.5 cm drifted slightly and that with 0.75 cm did not drift (Fig. S14e and S14f).


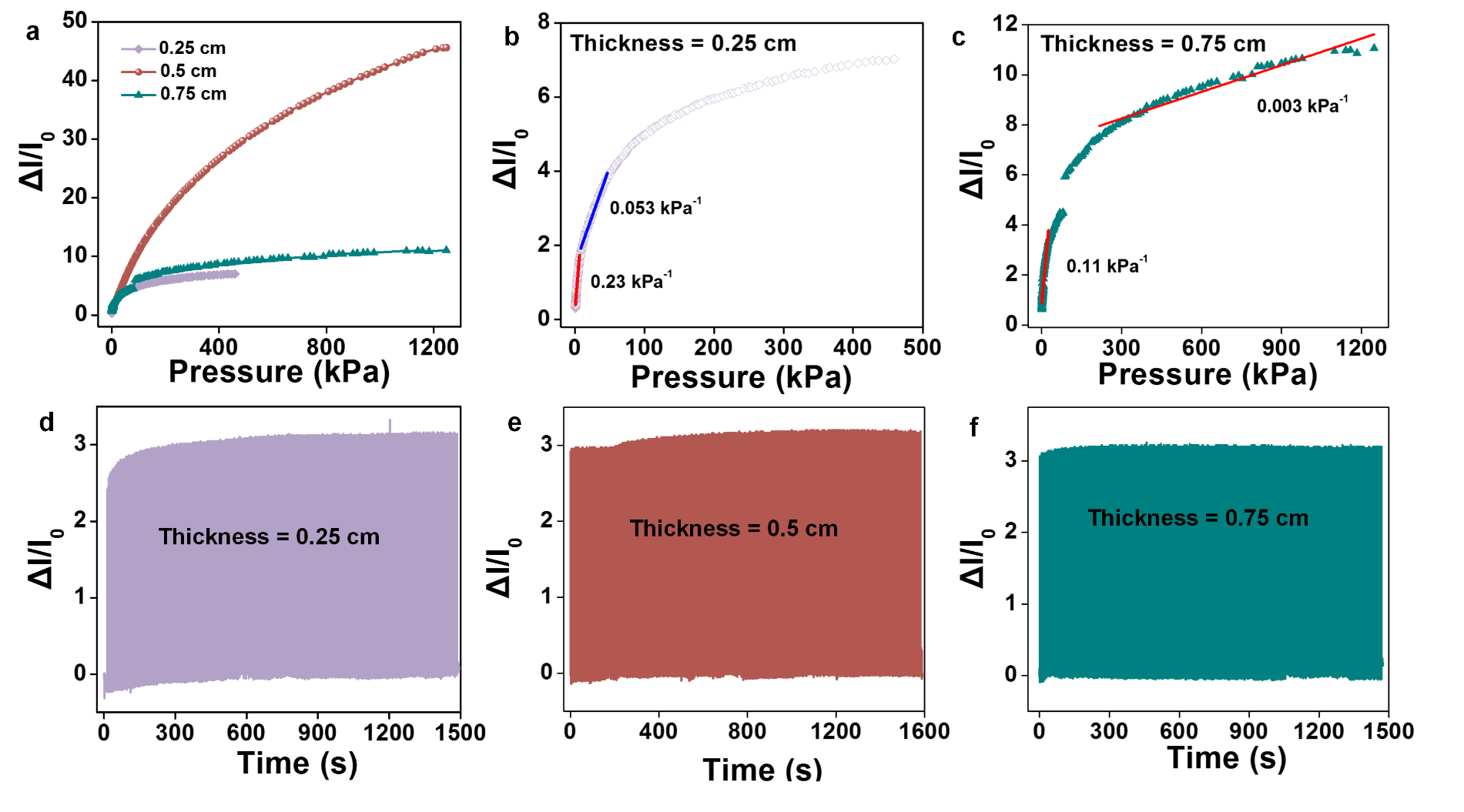


**Fig. S14** The sensing performances of MX/CS/PVDF-1 pressure sensor with different thickness sensitive materials. **a** The sensitivity of MX/CS/PVDF-1 pressure sensor with different thickness. **b** The linear fit curve of MX/CS/PVDF-1 pressure sensor with 0.25 cm. **c** The linear fit curve of MX/CS/PVDF-1 pressure sensor with 0.75 cm. Durability test under 500 loading/unloading cycles under17.68 kPa of MX/CS/PVDF-1 pressure sensor with **d** 0.25 cm, **e** 0.5 cm and **f** 0.75 cm

**
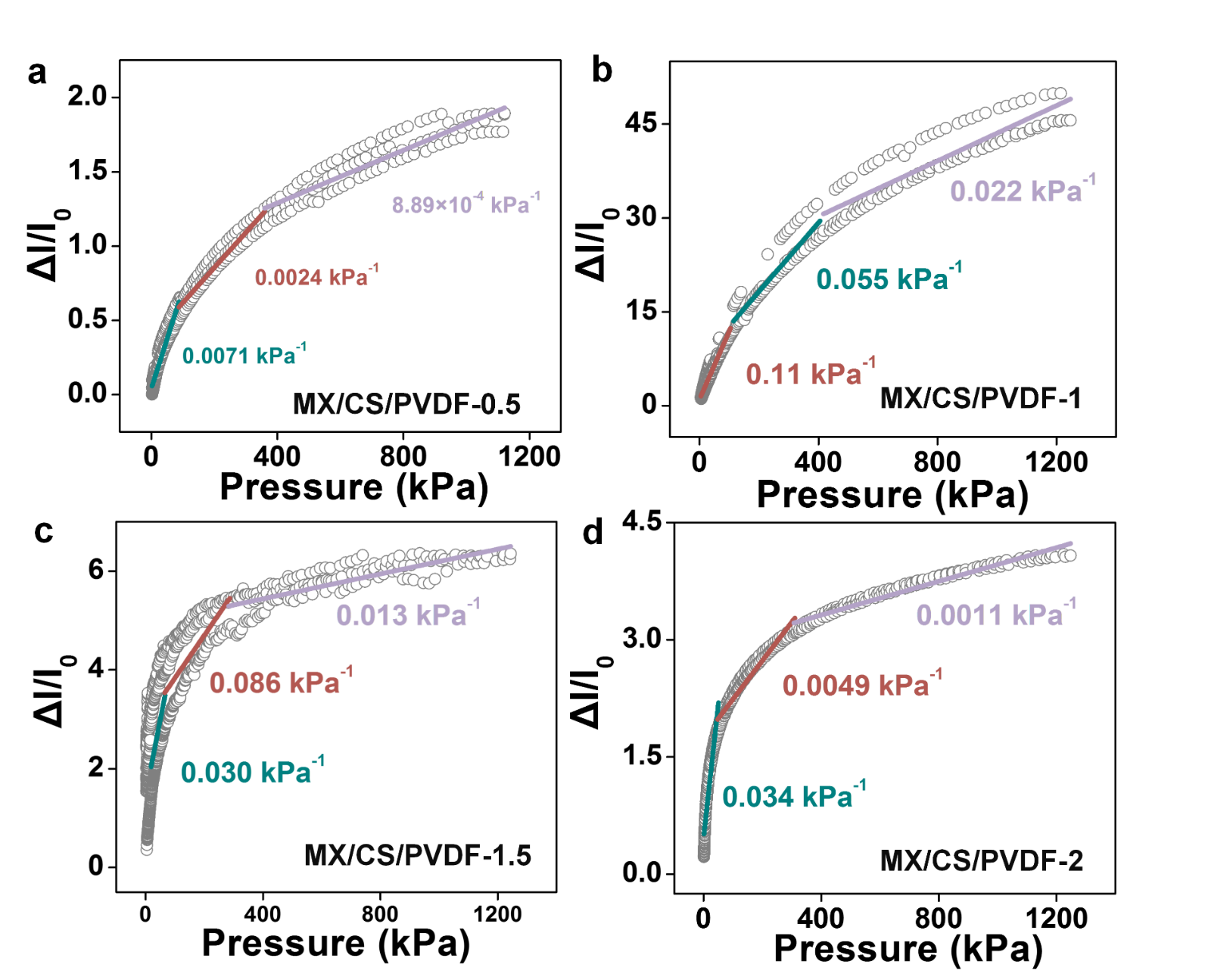
**

**Fig. S15** Response-pressure curves of **a** MX/CS/PVDF-0.5, **b** MX/CS/PVDF-1, **c** MX/CS/PVDF-1.5 and **d** MX/CS/PVDF-2 tested by three times

**
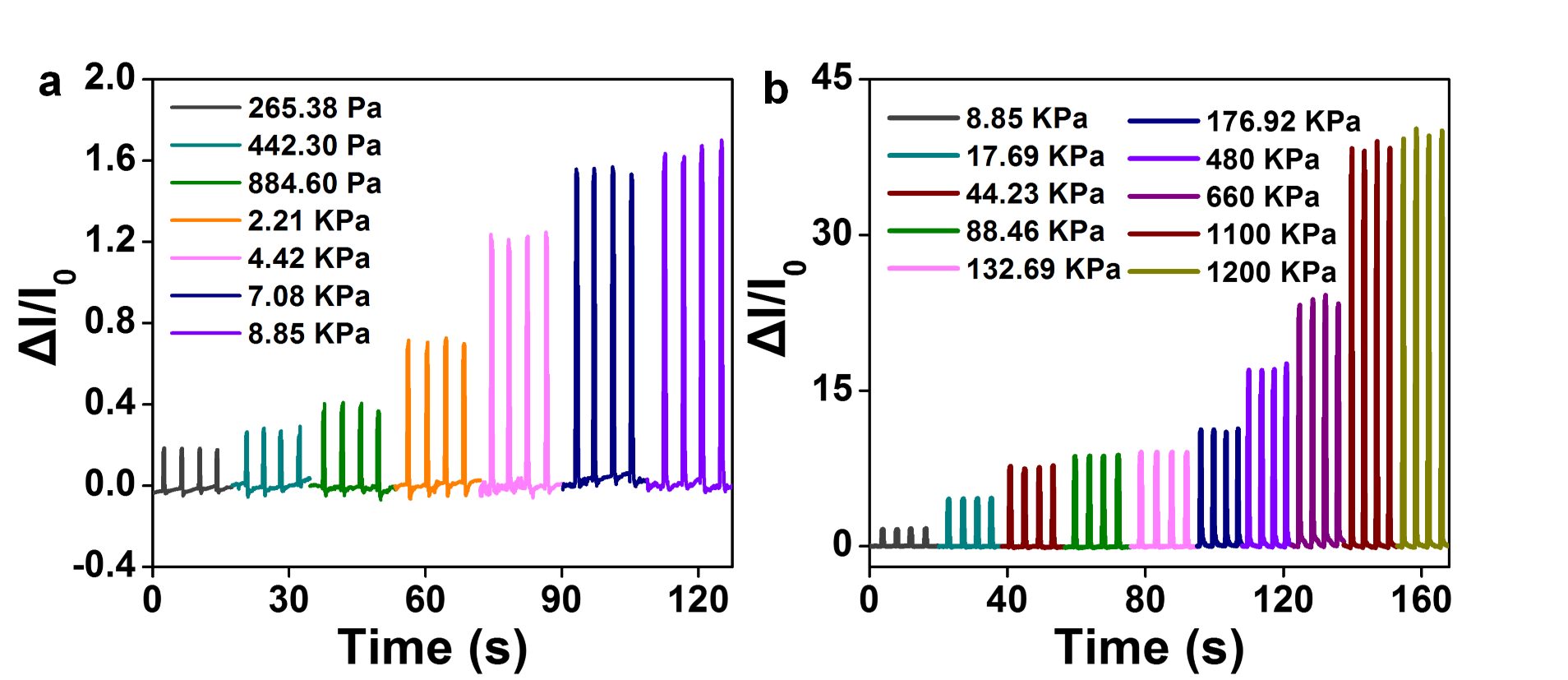
**

**Fig. S16** Relative changes in current of the MX/CS/PVDF-1 sensor **a** under low load pressure in the range of 265 Pa-8.85 kPa and **b** relative high load pressure in the range of 8.85 kPa-1200 kPa


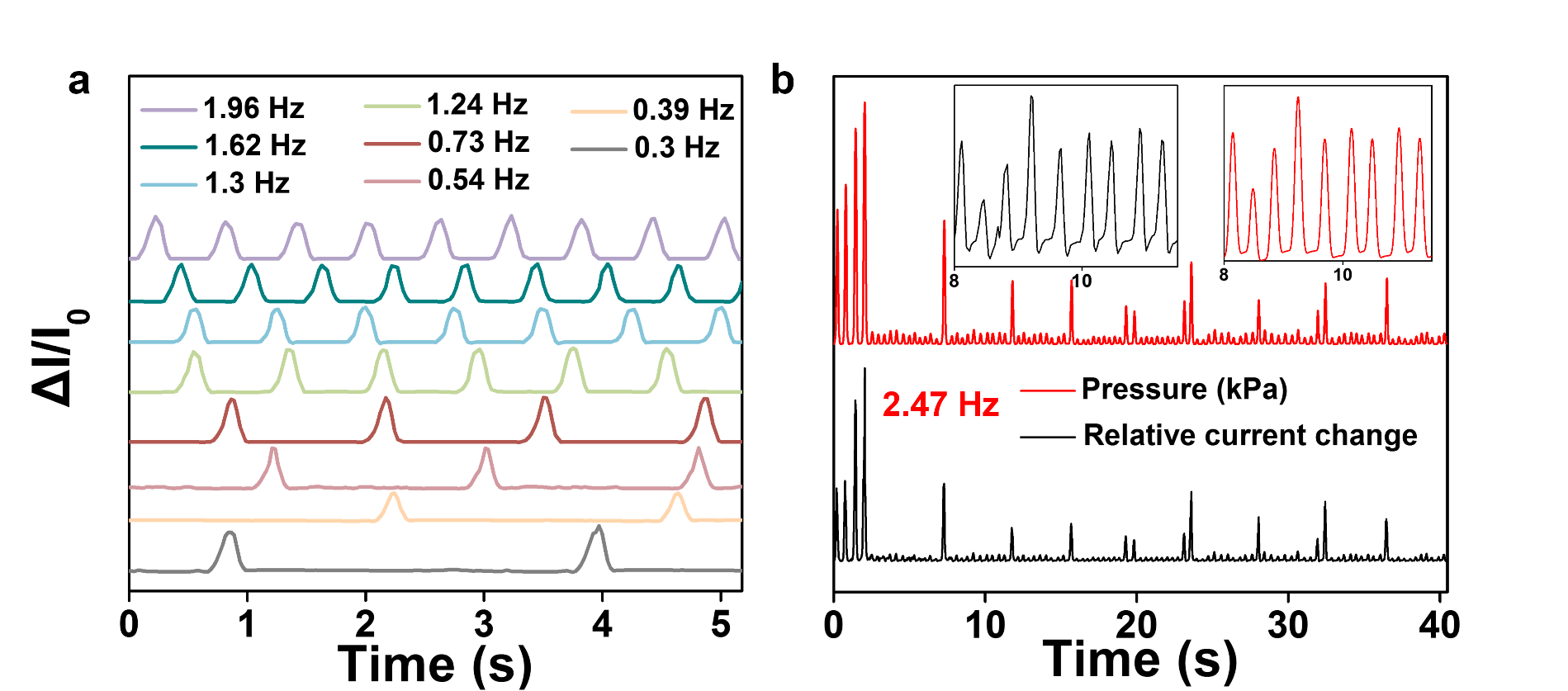


**Fig. S17** The variation of I-t curves with different running frequencies load pressure


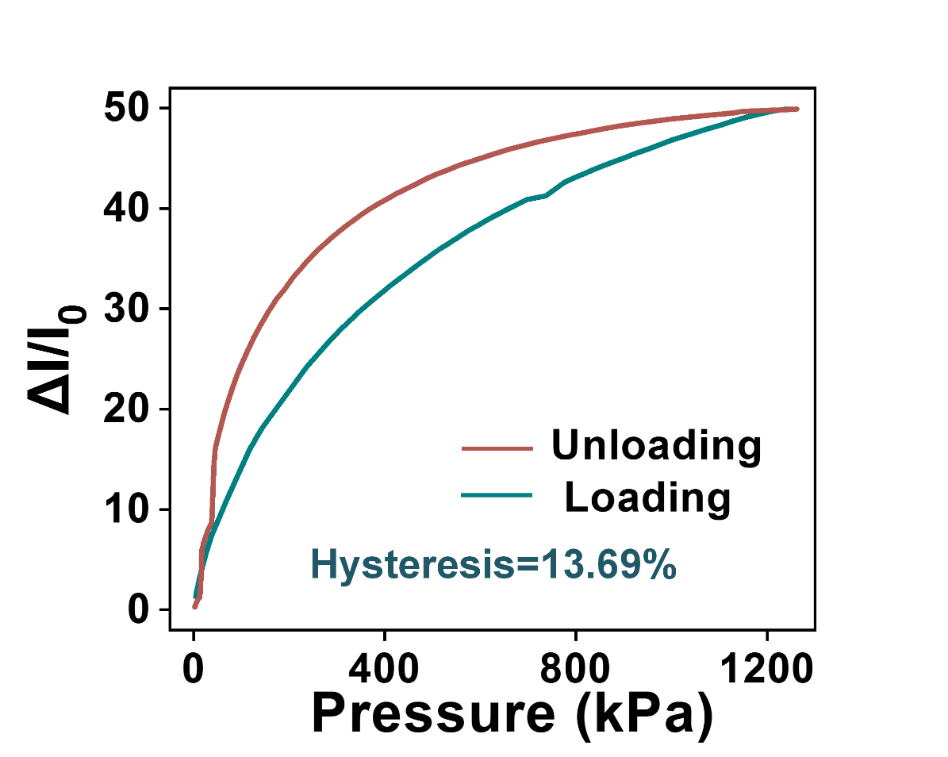


**Fig. S18** The hysteresis of MX/CS/PVDF-1-based pressure sensors under 1200 kPa


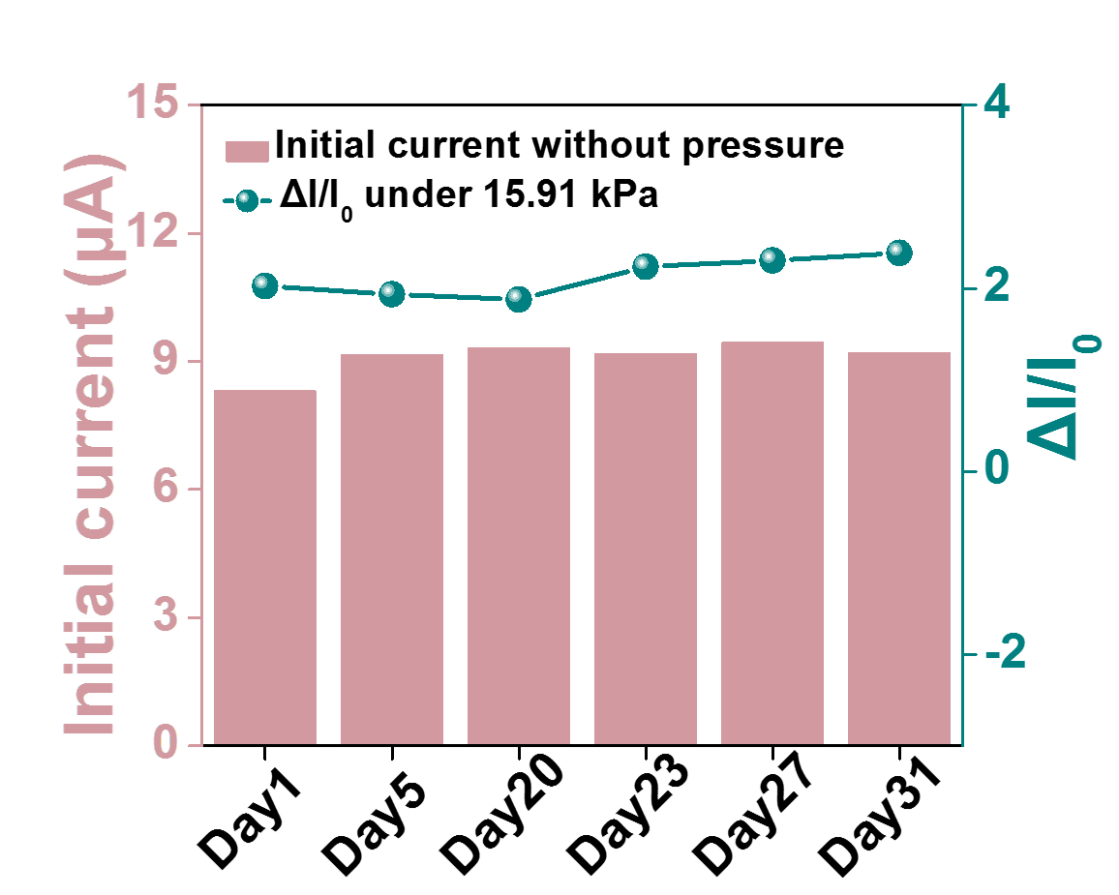


**Fig. S19** The initial current and relative current change of MX/CS/PVDF-1-based pressure sensors over a one-month period

**The stability of MX/CS/PVDF-1 pressure sensor at different ambient humidity and temperature:** To demonstrate the stability of MX/CS/PVDF-1 pressure sensor, the measurement under different ambient humidity and temperature was performed. The pressure sensor was placed in 33% relative humidity (RH), 59% RH, 85% RH, and 98% RH for 1 hour, where the relative humidity was provided by MgCl_2_, NaBr, KCl, and K_2_SO_4_ saturated solutions, respectively. Then the initial resistance and relative current change under pressure of 15.91 kPa were measured. As shown in Fig. S20a, the initial resistance and relative current change were stable. To simulate extreme ambient temperature, the pressure sensors were placed in a refrigerator at -20°C and an oven at 40°C for 5 days, respectively. Then the relative current change under pressure of 44.2 kPa was measured every day. As shown in Fig. S20b, c, the relative current change remained constant within limits.


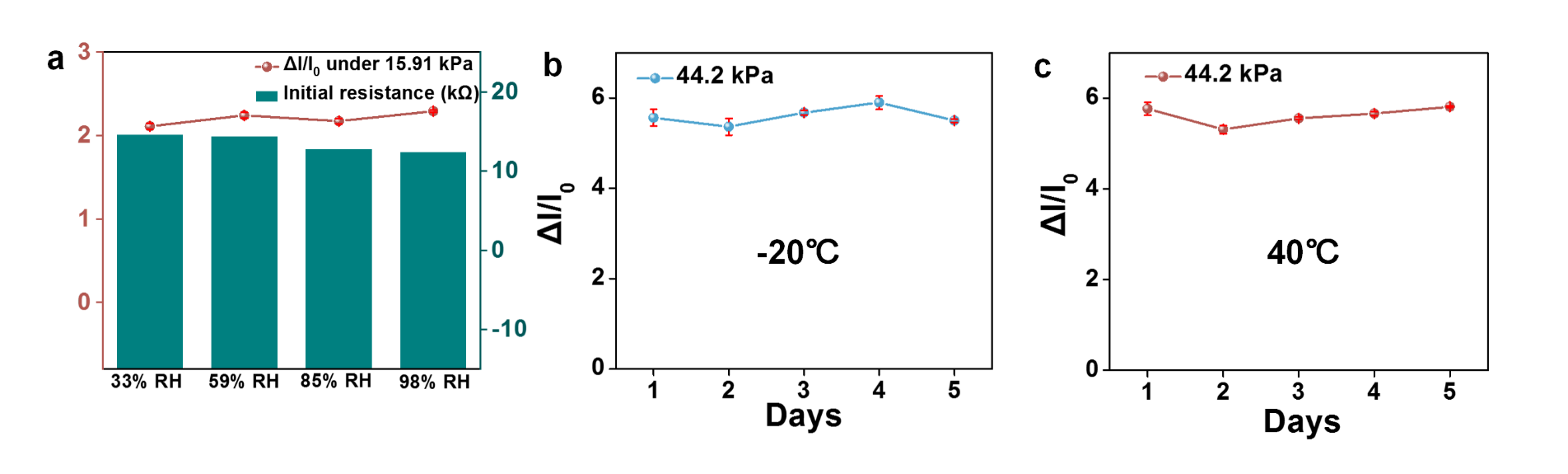


**Fig. S20** The stability of MX/CS/PVDF-1 pressure sensor under **a** varying ambient humidity and ambient temperature at **b** -20°C and **c** 40°C


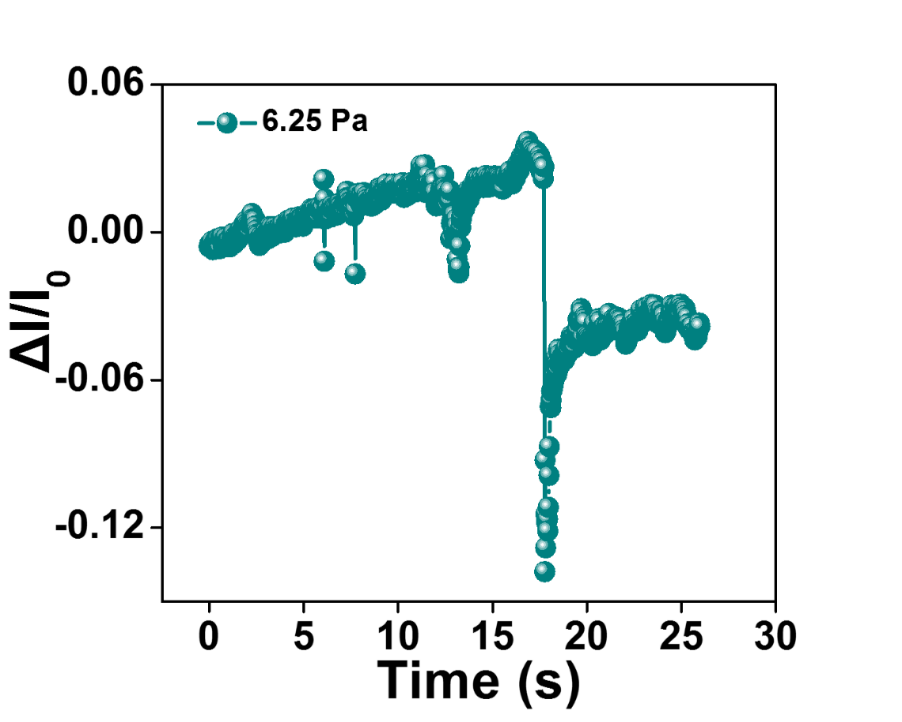


**Fig. S21** The detection limit of MX/CS/PVDF-1 pressure sensor

**The comparison between Ti_3_C_2_T_x_ MXene/CS/PVDF aerogel with random structure and laminated structure:** In terms of mechanical properties, the strain of the random-structured aerogel device exhibited irreversible increase after 100 cycles under 108 kPa pressure, indicating that the structural deformation of the random-structured aerogel device was not recoverable under higher stress (Fig. S22). In contrast, the laminated structure aerogel device maintained constant strain during cycle compression and demonstrated the potential for reversible deformation. Regarding the electrical properties, the laminated structure aerogel device demonstrated a higher current response under the same pressure and better linear relationship within the 100 kPa in comparison to the random-structured aerogel device (Fig. S23).


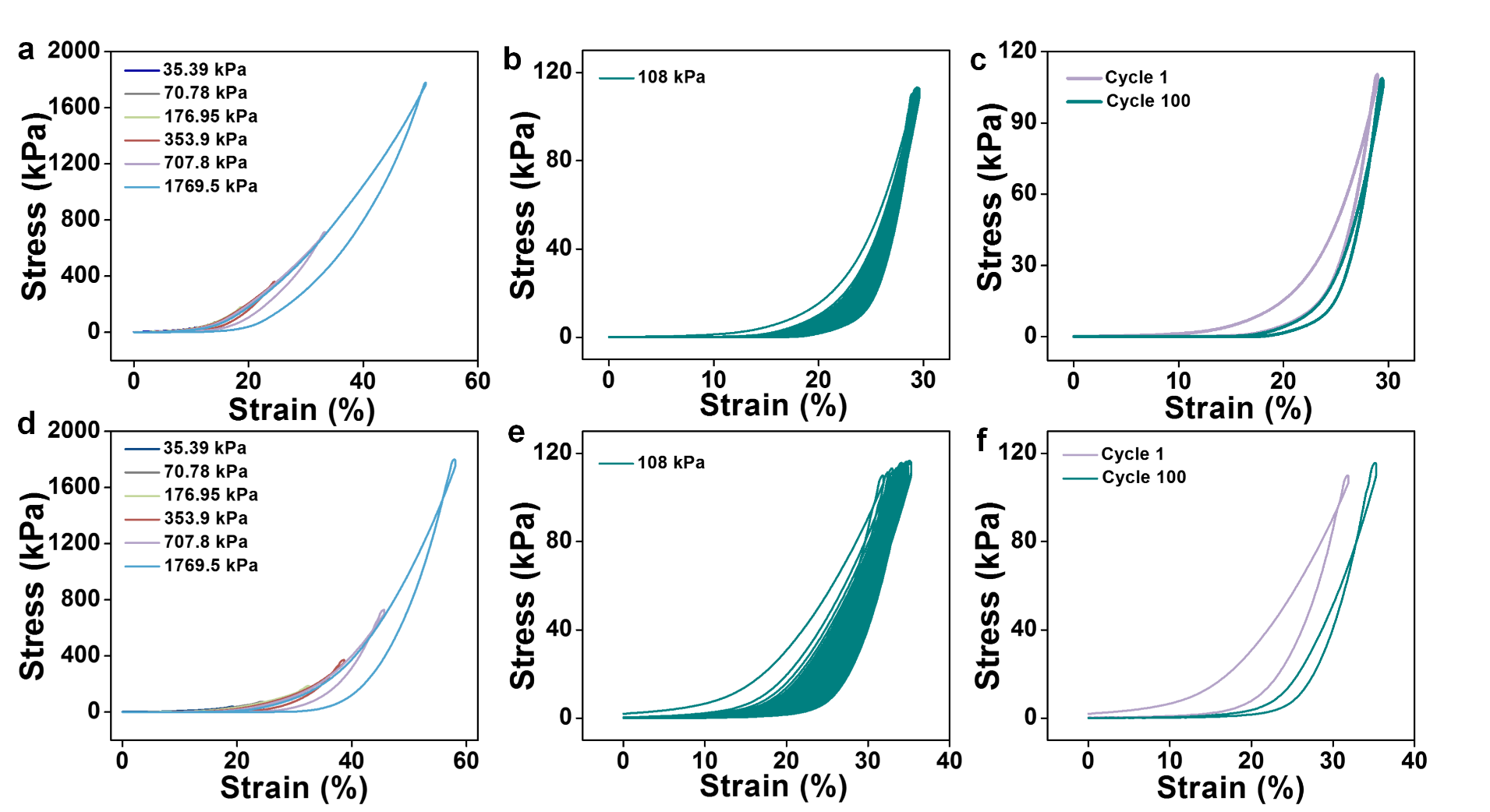


**Fig. S22** **a** Stress-strain cycle curves under different pressure loads, **b** stress-strain curves at 100 cycles and **c** stress-strain cycle curves of 1^st^ and 100^th^ of laminated structure MX/CS/PVDF-1-based pressure sensor. **d** Stress-strain cycle curves under different pressure loads, **e** stress-strain curves at 100 cycles and **f** stress-strain cycle curves of 1^st^ and 100^th^ of random structure MX/CS/PVDF-1-based pressure sensor


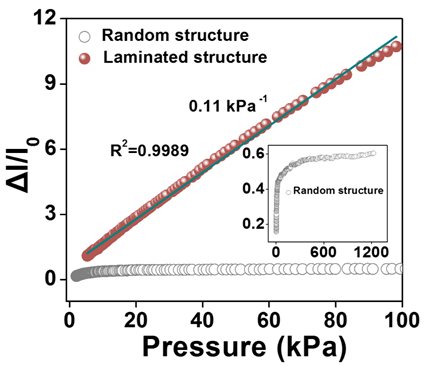


**Fig. S23** Response-pressure curves of random structure and laminated structure Ti_3_C_2_T_x_ MXene/CS/PVDF aerogel

**The effect of PVDF fiber length on the sensing performance:** To investigate the effect of PVDF fiber length on pressure sensing performance, the length of PVDF short fiber was controlled by regulating the breaking time of the as-spun PVDF fibers in the high-speed blender (5 min, 10 min, 15 min, 20 min) and measuring the response pressure curves and durability test of the pressure sensors containing PVDF short fibers with different breaking times.

As demonstrated in Fig. S24a, the sensitivity of the pressure sensors did not undergo a significant change with the breaking time of the PVDF fibers. However, the fiber breaking time had a significant influence on the durability. When the crushing time was 5 minutes, the initial current of the pressure sensor was drastically drifted under 500 repetitive cycles and the initial current was stabilized at a breakup time of 10 minutes or more (Fig. S24b). The explanation for this phenomenon was that with short breaking time, the fibers seemed too long to be uniformly suspended in the aerogel precursor solution due to gravity, and therefore cannot served to enhance the mechanical properties of the aerogel system, and also cannot demonstrated reversible deformation under greater stress.

The variations of the peaks of the relative current changes were caused by the unstable load pressure supplied by universal testing machine with high operating speed and had no relation to the stability of the pressure sensor.


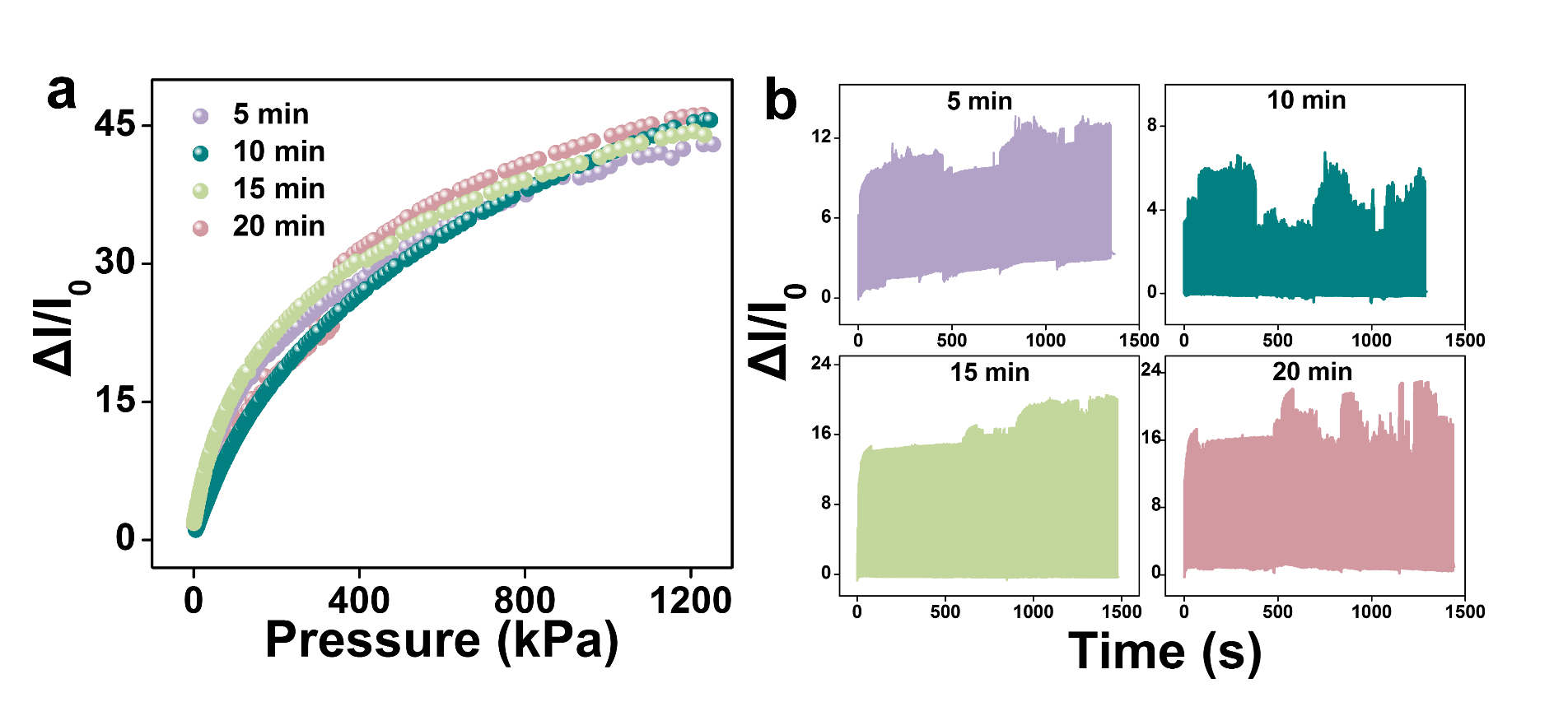


**Fig. S24** The effect of breaking time of PVDF fibers on device sensing performance. **a** The response-pressure curves and **b** durability test under 500 loading/unloading cycles of MX/CS/PVDF-1 with 5 min,10 min,15 min and 20 min breaking time


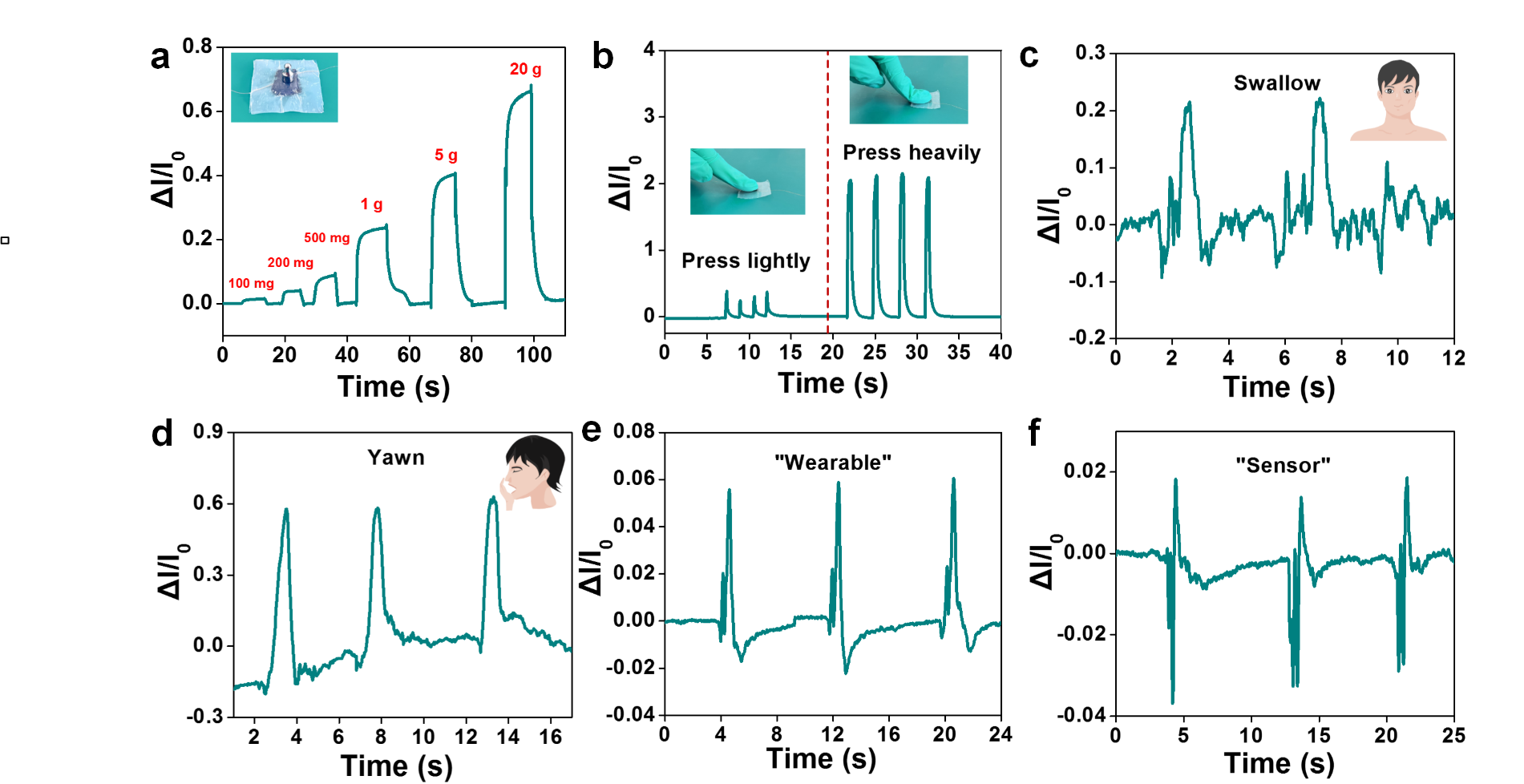


**Fig. S25 a** Relative current change of loading and unloading different weights from 100 mg to 20 g on the MX/CS/PVDF-1 sensor. **b** The relative current changes of manual press on the MX/CS/PVDF-1 sensor. The detection of human physiological signals: **c** swallow, **d** yawn, **e** say “wearable” and **f** say “sensor”


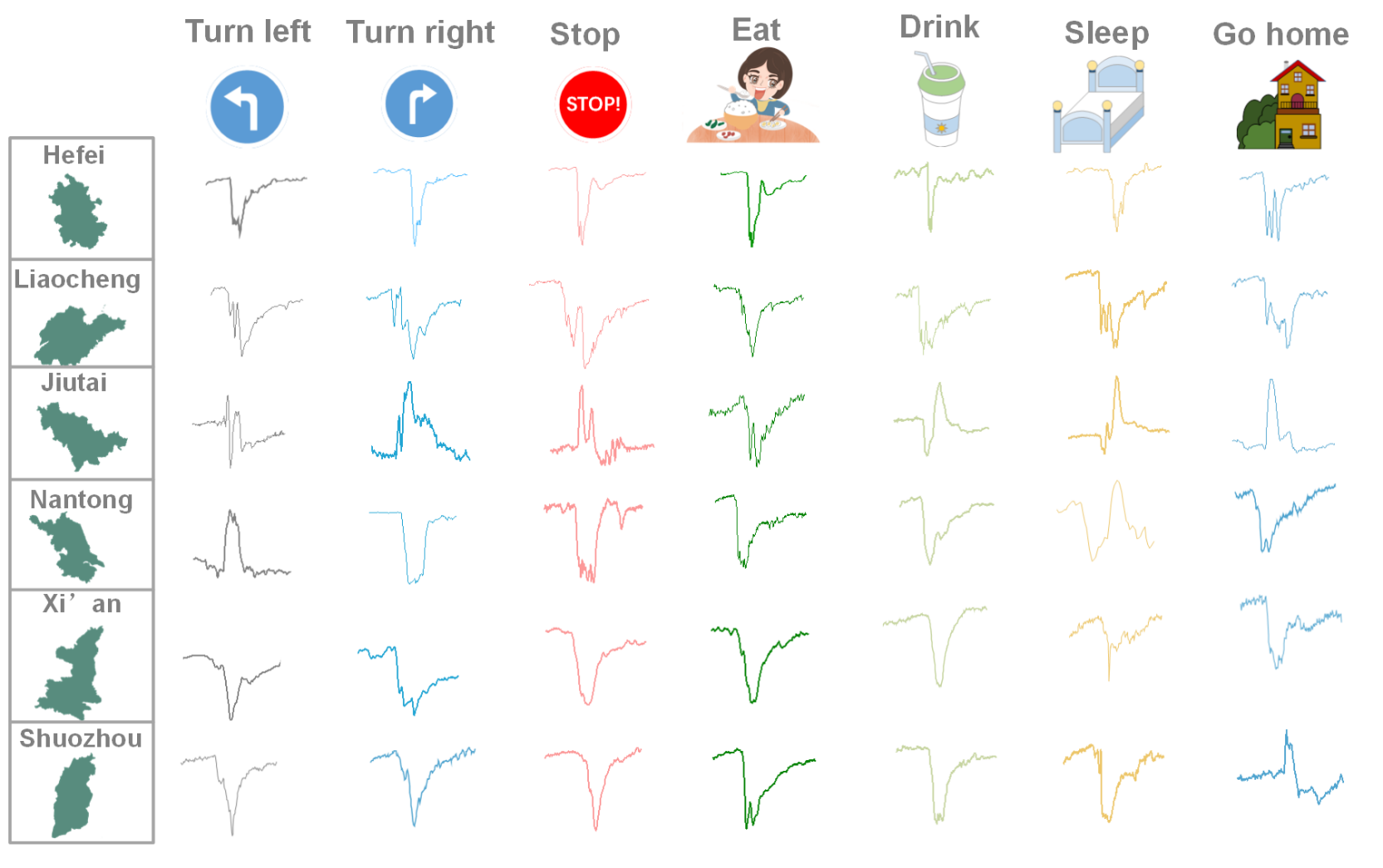


**Fig. S26** Waveform diagram of current signals generated by seven vocabularies in six Chinese dialects

**
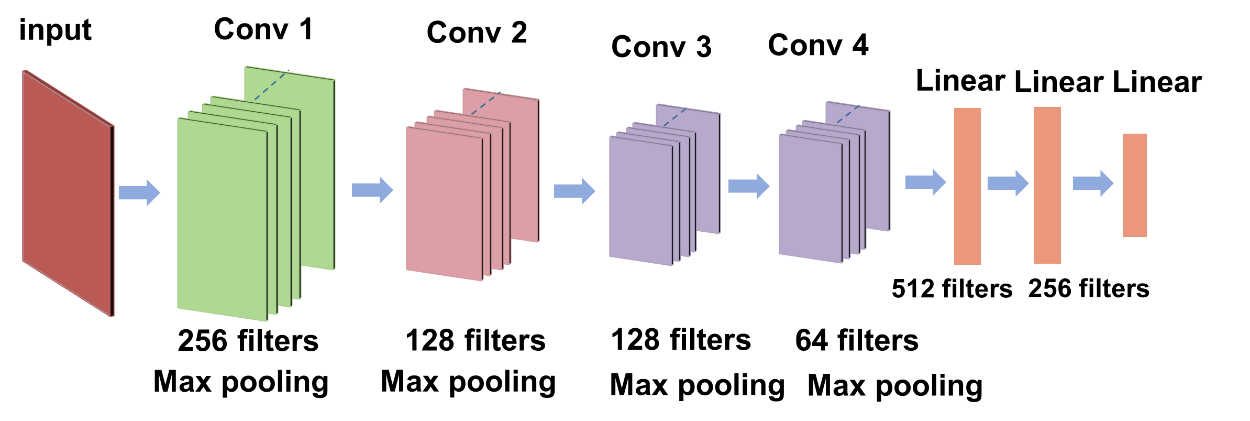
**

**Fig. S27** Schematic illustration of CNN

**Table S1** Comparison of the detection limit, maximum detection pressure, response/recovery time and hysteresis between the pressure sensors for speech recognition in this article and previous articles

| Material | Detection limit | Maximum detection pressure | Response/recovery time | Hysteresis | References | |
| --- | --- | --- | --- | --- | --- | --- |
| Molybdenum Disulfide/ hydroxyethyl cellulose/ polyurethane |  | 250 kPa | 120 ms |  | [S1] |  |
| Ti_3_C_2_T_x_@ poly(vinylidene fluoride-co-trifluoroethylene) | 72 Pa | 3.083 kPa | 16 ms |  | [S2] |  |
| Black phosphorous@ polyaniline | 500 Pa | 100 kPa | 200/210 ms | 3.43% | [S3] |  |
| Zwitterionic hydrogels |  | 3 kPa | 38 ms |  | [S4] |  |
| AuNWs/ Polydimethylsiloxane |  | 100 kPa | 50 ms |  | [S5] |  |
| Poly(3,4-ethylenedioxythiophene)/ poly-(styrenesulfonate)/cellulose nanocrystals |  | 100 kPa | 240/100 ms |  | [S6] |  |
| Carbon nanotubes/ polydimethylsiloxane |  | 80 kPa | 43/123 ms |  | [S7] |  |
| Multi-walled carbon nanotube/ polydimethylsiloxane | 20 Pa | 20 kPa | 25/50 ms | 8% | [S8] |  |
| Poly(vinylidene fluoride) acrylonitrile sodium p-styrenesulfonate acrylamide oxidized tannic acid N,N′-methylenebis(acrylamide) |  | 33.07 kPa | 24/45 ms | 13.80% | [S9] |  |
| Ti_3_C_2_T_x_ MXene/chitosan/polyvinylidene difluoride | 6.25 Pa | 1200 kPa | 72/72 ms | 13.69% | This work |  |

**References**

1. X. Chen, D. Zhang, H. Luan, C. Yang, W. Yan et al., Flexible pressure sensors based on molybdenum disulfide/hydroxyethyl cellulose/polyurethane sponge for motion detection and speech recognition using machine learning. ACS Appl. Mater. Inter. **15**, 2043 (2023). <https://doi.org/10.1021/acsami.2c16730>
2. L. Li, X. Fu, S. Chen, S. Uzun, A.S. Levitt et al., Hydrophobic and stable MXene-polymer pressure sensors for wearable electronics. ACS Appl. Mater. Inter. **12,** 15362 (2020). <https://doi.org/10.1021/acsami.0c00255>
3. J.V. Vaghasiya, C.C. Mayorga-Martinez, J. Vyskočil, M. Pumera, Black phosphorous-based human-machine communication interface. Nat Commun. **14,** 2 (2023). <https://doi.org/10.1038/s41467-022-34482-4>
4. S. Xu, J.-X. Yu, H. Guo, S. Tian, Y. Long et al., Force-induced ion generation in zwitterionic hydrogels for a sensitive silent-speech sensor. Nat Commun. **14,** 219 (2023). <https://doi.org/10.1038/s41467-023-35893-7>
5. S. Gong, X. Zhang, X.A. Nguyen, Q. Shi, F. Lin et al., Hierarchically resistive skins as specific and multimetric on-throat wearable biosensors. Nat Nanotechnol. **18**, 889 (2023). <https://doi.org/10.1038/s41565-023-01383-6>
6. H. Zhang, X. Chen, Y. Liu, C. Yang, W. Liu et al., Pdms film-based flexible pressure sensor array with surface protruding structure for human motion detection and wrist posture recognition. ACS Appl. Mater. Inter. **16,** 2554 (2024). <https://doi.org/10.1021/acsami.3c14036>
7. Y. Zhao, X. Lei, Z. Zeng, D. Guo, Y. Li et al., Highly sensitive flexible pressure sensors with hybrid microstructures similar to volcano sponge. ACS Appl. Mater. Inter. **15,** 54743 (2023). <https://doi.org/10.1021/acsami.3c14281>
8. B. Zhu, Z. Xu, X. Liu, Z. Wang, Y. Zhang et al., High-linearity flexible pressure sensor based on the gaussian-curve-shaped microstructure for human physiological signal monitoring. ACS Sens. **8,** 3127 (2023). <https://doi.org/10.1021/acssensors.3c00818>
9. Y. Shi, Y. Tian, Y. Guan, X. Kang, Y. Li et al., All-polymer piezoelectric elastomer with high stretchability, low hysteresis, self-adhesion, and UV-blocking as flexible sensor. ACS Appl. Mater. Inter. **15,** 43003 (2023). <https://doi.org/10.1021/acsami.3c09065>
